# Preoperative Beta‐Hydroxy‐Beta‐Methyl‐Butyrate Supplementation Reduces Mitochondrial Dynamics Proteins and Preserves Hepatic Mitochondrial Function After Partial Hepatectomy in Mice

**DOI:** 10.1111/apha.70204

**Published:** 2026-03-29

**Authors:** A. L. Vieira‐da‐Silva, M. V. Esteca, F. A. Silva, I. A. Divino, F. S. Carneiro, E. R. Ropelle, A. S. Torsoni, I. L. Baptista

**Affiliations:** ^1^ Laboratory of Cell and Tissue Biology (LaBCeT), School of Applied Sciences University of Campinas Limeira Brazil; ^2^ Laboratory of Metabolic Disorders (LabDiMe), School of Applied Sciences University of Campinas Limeira Brazil; ^3^ Laboratory of Molecular Biology of Exercise (LaBMEx), School of Applied Sciences University of Campinas Limeira Brazil

**Keywords:** liver mass recovery, liver metabolism, liver regeneration, mitochondrial network, mitochondrial protein, mtDNA content

## Abstract

**Aim:**

The liver exhibits a remarkable regenerative capacity, enabling this organ to maintain homeostasis even after significant injury. However, hepatic regeneration requires sufficient energy to sustain cellular hypertrophy and proliferation, thus ensuring efficient tissue repair. Therefore, dietary modulation of pathways regulating mitochondrial quality may enhance liver regeneration. This study aimed to investigate the effects of preoperative beta‐hydroxy‐beta‐methylbutyrate (HMB) supplementation on mitochondrial quality control pathways and its impact on the liver regeneration process in mice undergoing partial hepatectomy (PHx).

**Methods:**

Male C57BL/6J mice were supplemented with 600 mg/kg HMB via *gavage* for 10 days. On the 10th day, supplementation was discontinued, and the mice underwent a ⅔ liver resection. The subsequent 7 days have constituted the liver regeneration period. For a second injury induction, at the end of the 7th day of regeneration, acetaminophen (APAP) overdose was administered via *gavage*. We then analyzed several markers of mitochondrial quality and liver function.

**Results:**

Our results indicate that preoperative HMB supplementation modulates cell cycle progression, preventing excessive hepatic mass accumulation. Additionally, HMB regulates mitochondrial dynamics by decreasing Parkin, Mfn2, and DRP1 protein levels while increasing the mitochondrial markers VDAC2 and Tom20. Following a second injury from an APAP overdose, the HMB‐supplemented group demonstrated increased mtDNA content and enhanced mitochondrial capacity, both critical for effective tissue recovery.

**Conclusion:**

Our findings suggest that preoperative HMB supplementation preserves hepatic mitochondrial capacity after PHx.

## 1 Introduction

The regenerative capacity of the liver has been known since Ancient Greece and was first reported in the myth of Prometheus [1]. Given the central role played by this organ in human metabolism, liver regeneration evolutionarily acts as a protective mechanism against the loss of liver tissue, allowing the organ to continue to perform its functions even after suffering significant damage [2]. Partial hepatectomy (PHx) is a technique that consists of resecting ⅔ of the liver to induce organ regeneration without causing damage to the remaining tissue [3]. Liver resection is the most recommended medical treatment for cases of liver diseases, including malignant and benign tumors, and living donor liver transplantation [4].

Following the surgical excision of ⅔ of the liver, the remaining portion can rapidly regenerate and restore the organ to its original size, a process observed in rodents within 7 to 10 days post‐operation [5]. This hepatic regeneration involves both cell hypertrophy and proliferation. Cell hypertrophy is the initial response of hepatocytes to the loss of liver mass, occurring before they enter the cell cycle [6]. Usually, hepatocytes are quiescent in the G0 phase of the cell cycle. However, the cells' proliferative capacity is activated by stimuli from the injury, such as inflammation, and the subsequent action of cytokines and growth factors released by Kupffer cells during the priming phase [7]. With the aid of mitogens, hepatic cells enter the S phase and mitosis during the proliferation phase. Mitogens can be complete, possessing hepatotropic effects that stimulate DNA synthesis and cellular proliferation, such as various growth factors, or they can be auxiliary mitogens like bile acids, norepinephrine, insulin, estrogen, and serotonin, whose absence can delay regeneration [8]. Finally, when the liver returns to its normal size, negative factors such as interleukin (IL‐1 and IL‐6), members of the SOCS and TGF‐β family of proteins, and hepatocyte nuclear factor 4 alpha (HNF4‐α) return hepatic cells to quiescence and complete regeneration during the termination phase [9].

Hepatic regeneration is a process that varies depending on the energetic state of the remaining tissue [5]. In this context, mitochondrial function is crucial in the hepatic regenerative process, ensuring the proper energy supply to support cell proliferation and maintain hepatic functions after PHx [10]. However, a reduction in mitochondrial activity is often observed during liver regeneration due to the physiological stresses associated with surgery. Immediately after PHx, there is an overload in the portal venous system, which, in response, leads to arterial vasoconstriction to mitigate this excess. This vascular response creates a hypoxic environment following resection, compromising the functionality of liver mitochondria at a time when metabolic demands are particularly high [11]. Mitochondrial dysfunction is associated with an increase in mortality due to liver failure after PHx [12]. Therefore, maintaining mitochondrial integrity is critical not only for effective liver regeneration but also for postoperative survival. In this sense, dietetic modulation of mitochondrial population or mitochondrial dynamics in the liver may be considered a potential approach to assist in hepatic regeneration following ⅔ resection [13].

It has been demonstrated that administering carbohydrates such as glucose, fructose, or sorbitol post‐PHx inhibits liver regeneration in various injury models [14]. In contrast, lipids are an important energy source that supports tissue repair and should not be restricted [15]. Additionally, the regenerative process increases the demand for amino acids, and a low‐protein diet leads to reduced DNA synthesis [16]. Supplementation with branched‐chain amino acids (BCAA—valine, leucine, and isoleucine) appears to have favorable effects on tissue repair by stimulating protein synthesis and increasing glutamine production [17].

In this context, beta‐hydroxy‐beta‐methylbutyrate (HMB) is a metabolite derived from leucine. Dietary leucine is transaminated into alpha‐ketoisocaproic acid (KIC) in skeletal muscle tissue by BCAA aminotransferase (BCAT). Most KIC enters the bloodstream, and in the liver, about 5%–10% of KIC is converted by KIC dehydrogenase into HMB, which can then be transformed into HMG‐CoA and subsequently into cholesterol [18, 19, 20]. In other tissues, such as skeletal muscle, HMB affects metabolic pathways that stimulate protein synthesis and mitochondrial biogenesis while reducing protein catabolism, attenuating muscle fiber atrophy, and restoring grip strength in mice [21, 22, 23]. However, there needs to be more information about the effects of supplementation on the liver, where HMB is synthesized. The literature demonstrates that HMB supplementation might protect the liver against lipopolysaccharide (LPS)‐induced injury, potentially through mechanisms involving the enhancement of energy metabolism via AMP‐activated protein kinase (AMPKα) signaling [24]. The HMB treatment also inhibits the ubiquitin‐proteasome system and stimulates DNA synthesis in the early stages of liver regeneration in rats undergoing PHx [25]. However, the role of HMB over mitochondrial dynamics and its impact on liver regeneration is still unclear.

In this context, we hypothesized that preoperative supplementation with HMB could affect mitochondrial quality pathways and, consequently, the liver regeneration process in mice undergoing PHx. Our results indicate that HMB supplementation regulates mitochondrial dynamics by decreasing Parkin, Mfn2, and DRP1 protein levels while increasing VDAC2 and Tom20. Additionally, we found that HMB supplementation enhances tissue respiration and increases mtDNA content after a secondary injury induced by acetaminophen (APAP) overdose. Thus, our study suggests that HMB can improve mitochondrial capacity in the liver during hepatic regeneration in mice.

## 2 Materials and Methods

### 2.1 Animals

C57BL/6J male mice (12–14 weeks old, 20–29 g) were provided by the Multidisciplinary Center for Biological Research (CEMIB) and transported to the School of Applied Sciences of the University of Campinas (Limeira‐São Paulo, Brazil). The mice were kept under controlled conditions of a 12:12 h light–dark cycle in cages housed in cabinets with air exhaust and temperature and humidity control (20°C–26°C and 40%–60%, respectively), with free access to water and conventional feed (Nuvilab). All animal experiments were approved by the Ethics Committee on the Use of Animals of the University of Campinas (CEUA, protocol no. 5712‐1/2021 approved on March 18, 2021). The sample size was calculated based on the estimation of the mean of a simple random sample, using a 95% confidence interval (*Z* = 1.96), a standard deviation obtained from previous studies conducted by our group using the same intervention (*σ* = 0.065; unpublished data), and a precision level of 0.05. Using the formula *n* = (*Z*·*σ*)^2^/*E* ^2^, we obtained *N* = 6.49 or *N* = 7 animals per group.

#### 2.1.1 HMB Supplementation

HMB (Calcium β‐Hydroxy‐β‐Methylbutyrate, Alfa Aesar) was dissolved in drinking water at a dose of 600 mg/kg body weight/day, and 100 μL of this solution was administered orally (gavage) once a day [26]. The mice were allocated into three experimental groups: a group supplemented for 3 days (HMB 3d); a group supplemented for 10 days (HMB 10d); and a group supplemented for 10 days followed by a 7‐day discontinuation (HMB D/C). The control group received 100 μL of drinking water. After the respective supplementation periods, the animals were euthanized by anesthetic overdose, using a dose equivalent to three times the induction dose.

#### 2.1.2 Partial Hepatectomy

The mice received drinking water or HMB supplementation for 10 days. After this period, the supplementation was discontinued to assess the impact of pre‐surgical HMB on the tissue's regenerative capacity. On the 10th and final day of supplementation, the mice underwent PHx 1 h after the last HMB dose, following the protocol proposed by Mitchell & Willenbring [27]. A 3 cm abdominal incision was made to expose the liver. The left lobe was tied off with a non‐absorbable suture and completely removed using microsurgical scissors. The middle lobe was tied off above the gallbladder, and the portion below the knot was removed. The sham group underwent an opening of the abdominal cavity and manipulation of the liver with a flexible cotton‐tipped rod. The 7 days after surgery corresponded to the period of liver regeneration. At the end of this period, the mice were anesthetized in the afternoon using anesthesia equivalent to three times the induction dose. Blood was collected via cardiac puncture, and the liver was harvested, sectioned, and frozen in liquid nitrogen.

#### 2.1.3 Second Injury In Vivo Model

The mice received drinking water or HMB supplementation for 10 days and underwent partial hepatectomy. At the end of the 7th day of regeneration, APAP was administered via gavage at 400 mg/kg body weight. This dose was chosen to induce hepatotoxicity in C57BL/6 mice without causing mortality. The mice were euthanized after 24 h of APAP administration by anesthetic overdose using a dose equivalent to three times the induction dose. Blood was collected via cardiac puncture, and portions of the liver were preserved in liquid nitrogen.

### 2.2 Morphological Analysis

The right lobe of the liver was preserved using magnesium silicate to remove intrahepatic fluids. The sample was then covered with Tissue‐Tek O.C.T. Compound (Sakura) and immersed for 25 s in liquid nitrogen. Using a cryostat (Leica), the samples were cut at −25°C into 10 μm sections, adhering to the slides in serial sections. Tissues were cut through hematoxylin–eosin (H&E), Picrosirius red, and Oil‐Red.

#### 2.2.1 Hematoxylin and Eosin (H&E)

Slides containing histological sections were fixed in 95% alcohol for 2 min. Filtered hematoxylin was applied to the sections for 2 min, followed by a 3 min wash in running water. Eosin was then added to the sections for 5 min, and subsequent washes of 2 min each were performed in 70%, 80%, and 95% alcohol. Finally, the slides were sequentially washed in absolute alcohol I, absolute alcohol II, absolute alcohol III, alcohol‐xylol, xylol I, xylol II, and xylol III, each reagent being used for 1 min. The slides were mounted with a coverslip using Entellan (Sigma Aldrich). The images were acquired by light microscope (Leica) using a 20× and 40× objective. The lesion areas were counted using ImageJ software (Research Services Branch, National Institute of Mental Health). For this analysis, an average of 5 photographs of different regions and sections of the liver of each animal were used.

#### 2.2.2 Oil‐Red O Staining

The slides were washed using phosphate‐buffered saline (PBS) for 5 min. To prepare the Oil‐red O (ORO) stock solution, 1.25 g of the dye was dissolved in 200 mL of isopropyl alcohol. Subsequently, 18 mL of ORO was combined with 12 mL of distilled water, creating the working solution. The slides were then immersed in the working solution for 30 min. Following this period, hematoxylin was applied for an additional 5 min. The slides were subsequently washed in running water for 30 min and ultimately sealed with a coverslip and gelatine. The images were acquired by a light microscope (Leica) using a 20× and 40× objective. The area of lipid droplets was measured using ImageJ software (Research Services Branch, National Institute of Mental Health). In this analysis, an average of 5 photographs per animal from different regions and sections of the liver was used.

#### 2.2.3 Immunofluorescence

Slides containing serial sections were washed in PBS and fixed with 4% paraformaldehyde (PFA) dissolved in PBS for 10 min, with subsequent permeabilization in phosphate‐buffered saline with 0.1% Tween 20 (PBS‐T) solution. The slides were immersed in a citric acid buffer at 95°C for 5 min and then underwent a wash in PBS‐T. A blocking solution containing 3% bovine serum albumin (BSA), 5% serum from a mouse (Sigma Aldrich), and PBS‐T was added to each slide for 40 min. Then, another solution with 3% BSA, 5% normal goat serum (R&D Systems), and PBS‐T was added for 30 min. The slides were rewashed for 10 min in PBS‐T and were incubated overnight at 4°C in a humid chamber in a solution containing 3% BSA, 1% glycine, PBS‐T, and the primary antibodies (see Table S1). After two washes in PBS‐T, the slides were incubated at room temperature with low light for 1 h with a solution containing 1% BSA, PBS‐T, and the secondary antibodies (see Table S2). The slides were briefly washed in PBS‐T and received Sudan Black B solution (0.3% Sudan Black B in 70% ethanol) for 1.5 min. The excess solution was removed with 50% alcohol and washed with PBS. Finally, the slides were mounted with coverslips and sealed with VECTASHIELD Antifade Mounting Medium with DAPI (Vector Laboratories). The images were acquired by a light microscope (Leica) using a 20× and 40× objective. We controlled the exposure time for immunofluorescence using the negative control's background signal as a base. The Ki‐67 positive cells were counted manually with the aid of ImageJ software (Research Services Branch, National Institute of Mental Health). For the count, the evaluator was blinded. In this analysis, an average of 5 photographs per animal from different regions and sections of the liver were used. For the quantification of the Laminin‐stained area, ImageJ software (Research Services Branch, National Institute of Mental Health) was used. The fluorescence channels were split, and a fixed threshold range of 50–255 was applied to the red channel. As in the previous analysis, an average of 5 images from distinct liver regions was evaluated for each animal.

### 2.3 Biochemical Analysis

Biochemical analyses were carried out using blood collected by cardiac puncture at the time of euthanasia with the mice in the fed state. Analyses of alanine aminotransferase (ALT), aspartate aminotransferase (AST), glucose, cholesterol, triglycerides, and albumin were carried out using the enzymatic colorimetric test following the manufacturer's instructions (K049, K048, K083, K117, K082, and K040, Bioclin). Insulin was analyzed using the ELISA technique (EMINS, ThermoFisher Scientific), following the manufacturer's recommendations.

### 2.4 Western Blotting

The liver samples were homogenized in extraction buffer (0.625% Nonidet P‐40; 0.625% [w/v] sodium deoxycholate; 0.00625 M sodium phosphate, pH 7.2; 1 mM EDTA, pH 8; and 1% phosphatase inhibitor [Sigma‐Aldrich] and protease inhibitor [Sigma‐Aldrich]) at the ratio of 0.1 g tissue to 100 μL of buffer and centrifuged at 15 000 *g* for 10 min. The SDS‐PAGE gel was prepared with a gradient concentration of 4% to 20%, and 20 μg of protein was applied. The electrophoresis run was performed with subsequent transfer to the nitrocellulose membrane, which was stained with Ponceau Red (Sigma‐Aldrich). After a 1 h block with a 5% milk solution in Tris buffer saline with 0.1% Tween 20 (TBS‐T), the membranes were incubated overnight at 4°C in a 5% BSA solution in TBS‐T containing the primary antibody (see Table S1). The membranes were washed with TBS‐T and incubated with peroxidase‐conjugated secondary antibodies (see Table S2) for 1 h at room temperature. Images were acquired using Western Blotting Pierce ECL (ThermoFisher Scientific) and developed using the ChemiDoc system (Bio‐RAD), which allows the detection of the chemiluminescence signal. The quantitative densitometric analysis of the samples was conducted using ImageJ software.

### 2.5 High‐Resolution Respirometry

The oxygen consumption was determined using high‐resolution respirometry analysis (NextGen‐O2k). A 2 μg sample was used, equivalent to 20 μL of MiR05 (110 mM sucrose, 60 mM K‐lactobionate, 0.5 mM EGTA, 3 mM MgCl_2_, 20 mM taurine, 10 mM KH_2_PO_4_, 20 mM HEPES, pH 7.1, and 0.1% BSA) with the macerated tissue adjusted to 1 μg/10 μL. The following substrate/inhibitor dosages were used for the run: Malate (2.5 μL at 0.4 M) + Glutamate (10 μL at 2 M); ADP (10 μL at 0.5 M); Succinate (20 μL at 1 M); Oligomycin (1 μL at 5 mM); FCCP (3 μL in titration at 1 mM); Rotenone (1 μL at 1 mM) + Antimycin (1 μL at 5 mM).

### 2.6 Mitochondrial DNA (mtDNA) Quantification

A 10 mg portion of liver tissue was used for DNA extraction. The samples were incubated for 3 h at 55°C in a lysis buffer containing proteinase K (100 mM NaCl; 10 mM EDTA, pH 7.4; 0.5% (w/v) SDS; 20 mM Tris–HCl, pH 7.4; and 0.2 mg/mL proteinase K). 100 μg/mL RNase A was added to degrade RNA, followed by a 30 min incubation at 37°C. Subsequently, 250 μL of 7.5 M ammonium acetate and 600 μL of isopropanol were added, and the samples were centrifuged at 15 000 *g* for 10 min at 4°C to pellet the DNA. The DNA pellet was washed with 70% ethanol, dried, and resuspended in TE buffer (10 mM Tris–HCl; 1 mM EDTA; pH 8.0). For qPCR, 40 μg of DNA was used, with PCR cycling conditions as follows: initial denaturation at 95°C for 10 min, followed by 45 cycles of denaturation at 95°C for 10 s, annealing at 60°C for 10 s, and extension at 72°C for 30 s. A melting curve analysis was performed with a step at 95°C for 5 s, annealing at 66°C for 1 min, and a gradual increase in temperature up to 97°C for fluorescence acquisition. The primer sequences used were as follows:

ND1 FWD: 5′‐CCGCAAGGGAAAGATGAAAGAC‐3′;

ND1 REV: 5′‐TCGTTTGGTTTCGGGGTTTC‐3′.

HK2 FWD: 5′‐GCCAGCCTCTCCTGATTTTAGTGT‐3′;

HK2 REV: 5′‐GGGAACACACCCGACCTCTTCTGG‐3′.

### 2.7 Statistical Analyzes

For the construction of representative graphs, the mean ± standard error of the mean (SEM) was employed in cases where multiple experiments contributed to the total sample size. In contrast, the mean ± standard deviation (SD) was applied for the joint analysis of all samples. The statistical significance of differences between the two groups was ascertained using the Student's *t*‐test. To assess data distribution, the Shapiro–Wilk normality test was performed. In scenarios involving comparisons among four distinct groups, one‐way analysis of variance (ANOVA) was employed, followed by Bonferroni's post hoc test. The threshold for statistical significance was established at *p* < 0.05. All statistical analyses were performed using GraphPad Prism 8 software.

## 3 Results

### 3.1 HMB Supplementation Per Se Does Not Significantly Modulate Markers of Protein Synthesis and Mitochondrial Biogenesis in the Intact Liver

**FIGURE 1:**
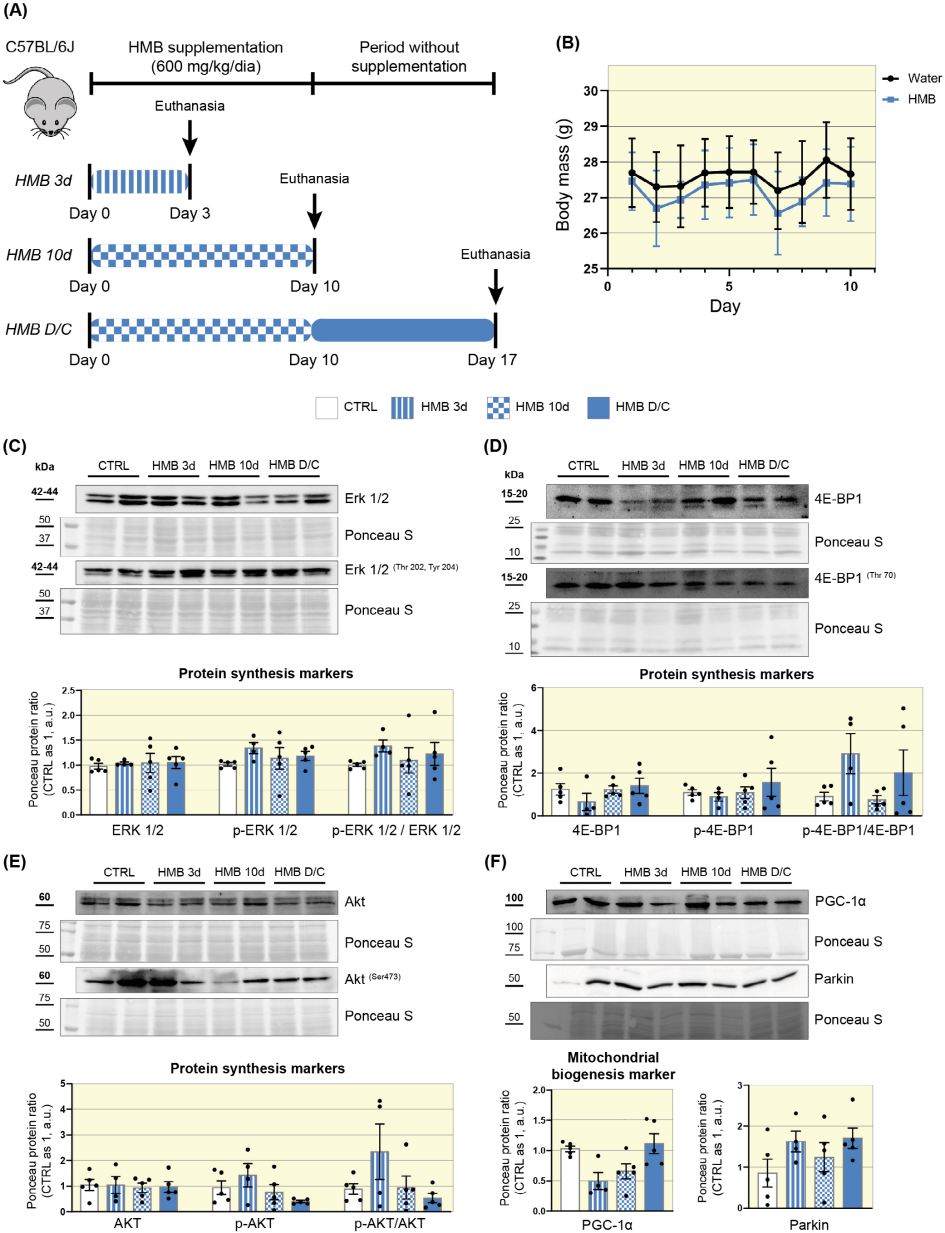
HMB supplementation per se does not significantly modulate markers of protein synthesis and mitochondrial biogenesis. (A) Diagram of supplementation timeline. (B) Graph showing the body weight of the groups during the experimental period (*n* = 5). (C) Representative Western blot images and graph showing the results of densitometry analysis of ERK 1/2, p‐ERK 1/2, and the ERK 1/2/p‐ERK 1/2 ratio (*n* = 4–5). (D) Representative Western blot images and graphs showing the results of densitometry analysis of 4E‐BP1, p‐4E‐BP1, and the p‐4E‐BP1/4E‐BP1 ratio (*n* = 4–5). (E) Representative Western blot images and graph showing the results of densitometry analysis of Akt, p‐Akt, and the p‐Akt/Akt ratio (*n* = 4–5). (F) Representative Western blot images and graph showing the results of densitometry analysis of the protein content of PGC‐1α and Parkin (*n* = 4–5). Data are presented as mean ± SEM. CTRL is set as 1; Ponceau S was used as a loading control.

Oral HMB supplementation demonstrates potential for modulating mitochondrial biogenesis and stimulating protein synthesis pathways, primarily in skeletal muscle. However, although HMB is synthesized in the liver, its effects on this tissue have been little investigated. Therefore, we began our study by evaluating whether HMB supplementation per se could modulate key markers of protein synthesis and mitochondrial biogenesis in an intact liver. To this end, the mice were divided into three study groups: a group supplemented for 3 days (HMB 3d), a group supplemented for 10 days (HMB 10d), and a group supplemented for 10 days followed by a 7‐day discontinuation (HMB D/C) (Figure 1A).

All study groups showed equivalent body mass after different supplementation times (Figure 1B). We assessed the activation of protein synthesis pathways by measuring the phosphorylation of extracellular signal‐regulated kinases (Erk^(Thr202 and Tyr204)^), eukaryotic translation initiation factor 4E binding protein 1 (4E‐BP1^(Thr70)^), and protein kinase B (Akt^(Ser473)^). Interestingly, we have not found differences between the studied groups (Figure 1C–E). We also analyzed the stimulation of mitochondrial biogenesis by quantifying the content of peroxisome proliferator‐activated receptor gamma coactivator 1‐alpha (PGC‐1α), the main transcription factor responsible for activating this pathway. We did not find significant effects on this protein content (Figure 1F). Finally, we assessed Parkin content, an E3 ligase associated with mitophagy and mitochondrial biogenesis, and found no significant differences (Figure 1F). These data indicate that HMB supplementation per se for different periods does not significantly affect markers associated with protein synthesis and mitochondrial biogenesis in the intact liver.

### 3.2 Preoperative Supplementation With HMB Prevents the Accumulation of Liver Mass During the Final Phase of the Regenerative Process

**FIGURE 2:**
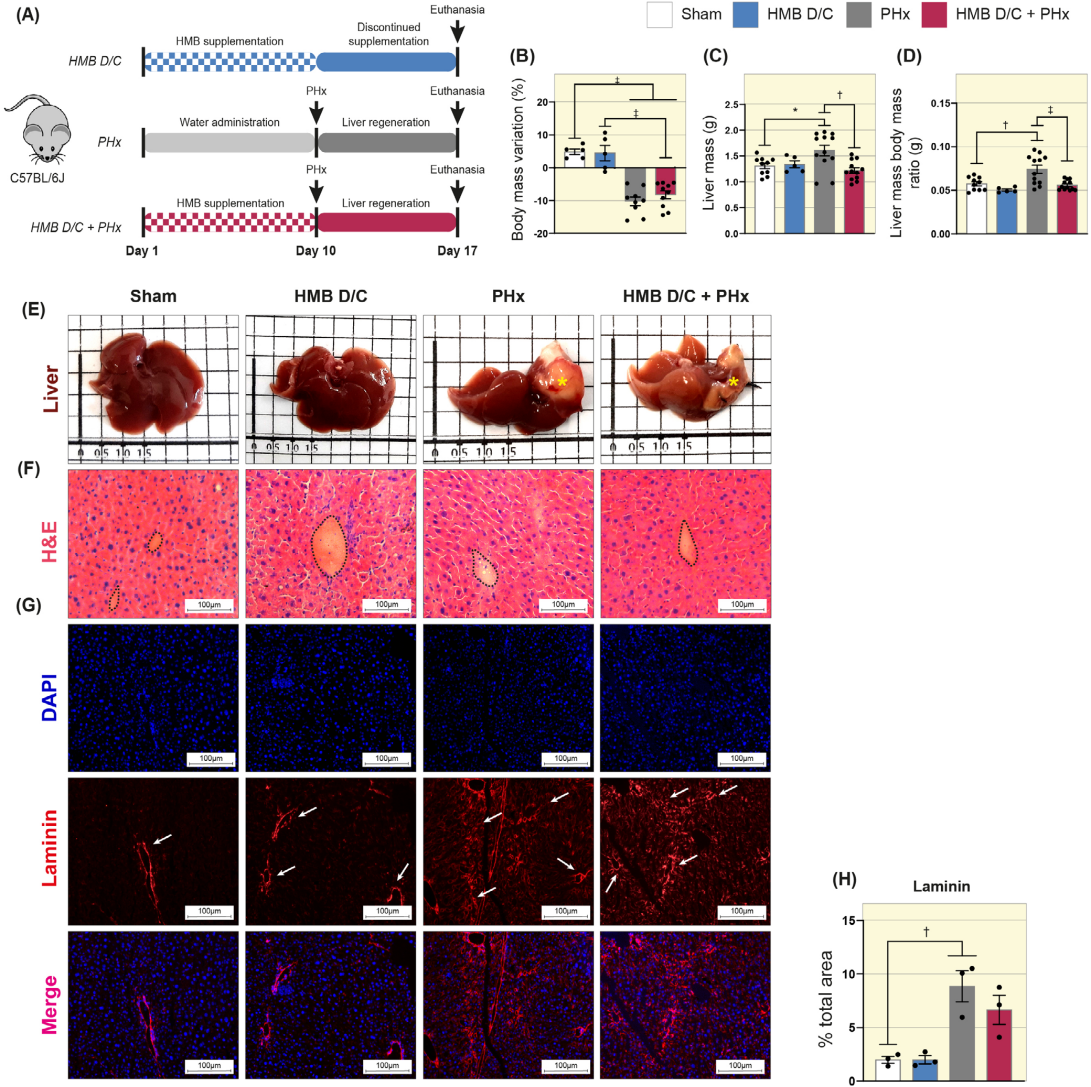
Preoperative supplementation with HMB prevents the accumulation of liver mass during the final phase of the regenerative process. (A) Diagram illustrating the period of HMB supplementation before liver regeneration in mice subjected to PHx. (B) Graph showing body mass variation (*n* = 5–17). (C) Graph showing liver mass (*n* = 5–12). (D) Graph showing liver mass–body mass ratio (*n* = 5–12). (E) Liver anatomopathological representative images of the different study groups. The asterisks indicate the growth of a hepatic mass. (F) Representative images of liver cross‐sections stained with H&E. The dashed lines mark the central veins. Images acquired with a 20× objective. Scale bar: 100 μm. (G) Representative images of Laminin immunofluorescence in mouse liver cross‐sections. Images were acquired with a 20× objective. Scale bar: 100 μm. Arrows point to the Laminin‐stained area. (H) Graph showing the total Laminin‐stained area (*n* = 3). Data are presented as mean ± SEM. The solid line represents One‐way ANOVA followed by Bonferroni's post hoc test: *p < 0.05, ^†^ *p* < 0.01, ^‡^ *p* < 0.001. Sham is set as 1.

HMB supplementation usually has pronounced effects in stressful conditions such as sarcopenia, cancer‐induced cachexia, and aging, especially in the prevention of muscle mass loss. Therefore, we investigated its possible effects under conditions of liver stress. To this end, the mice underwent 7 days of liver regeneration, after which they were euthanized with an anesthetic overdose (Figure 2A).

Initially, we evaluated the impact of HMB supplementation and the surgery on body mass, liver mass, and tissue morphology. Both groups undergoing ⅔ resection decreased their body weight compared to the sham group, with HMB not exhibiting protective effects against body mass loss in the HMB D/C + PHx group (Figure 2B). We observed a significant elevation in liver mass (PHx 1.61 vs. HMB D/C + PHx 1.22 a.u., Figure 2C) and in liver mass‐body mass ratio (PHx 0.07 vs. HMB D/C + PHx 0.05 a.u., Figure 2D) in the PHx group, which was not observed in the HMB D/C + PHx group, which maintained its liver mass and liver mass‐body mass ratio at levels similar to those of the sham group. Macroscopic anatomical images of the liver revealed the growth of a hepatic mass near the surgical incision site, particularly in the PHx group (Figure 2E). Histopathological evaluation of this region showed a pronounced inflammatory infiltrate in the HMB D/C + PHx group, as well as marked fat accumulation in the PHx group (Figure S1A). Our Western blot analysis did not show significant differences in the levels of inflammatory markers, including superoxide dismutase 2 (SOD2, associated with protection against oxidative stress), nuclear factor kappa B (NF‐κB, a transcription factor regulating genes involved in immune and inflammatory responses), interleukin 6 (IL‐6, a pro‐inflammatory cytokine), and interleukin 10 (IL‐10, an anti‐inflammatory cytokine) (Figure S1B–F).

We further examined the morphology of the remaining tissue. Histological assessment using H&E staining did not reveal any significant alterations in liver tissue morphology among the study groups (Figure 2F). We observed a reduction in the percentage of total Picrosirius Red‐stained area in both groups subjected to ⅔ hepatectomy compared with the sham group (Figure S1G). We also investigated the content of laminin, a non‐collagenous connective tissue glycoprotein that is a major constituent of basement membranes, using immunofluorescence. Interestingly, we detected a significant increase in the total Laminin‐stained area in the PHx group compared with the sham group (Sham 1.99 vs. PHx 8.85 a.u., Figure 2G,H).

Together, these findings suggest that HMB supplementation may attenuate the growth of hepatic masses at the excision site, as observed in the macroscopic anatomical analysis, thereby maintaining liver weight at levels comparable to those of the sham group. Concurrently, ⅔ hepatectomy was associated with a reduction in Picrosirius Red–stained area and an increase in laminin content, with no significant effect attributable to HMB supplementation.

### 3.3 Prior Supplementation With HMB Reduces Serum Levels of Hepatic Damage Markers After PHx

**FIGURE 3:**
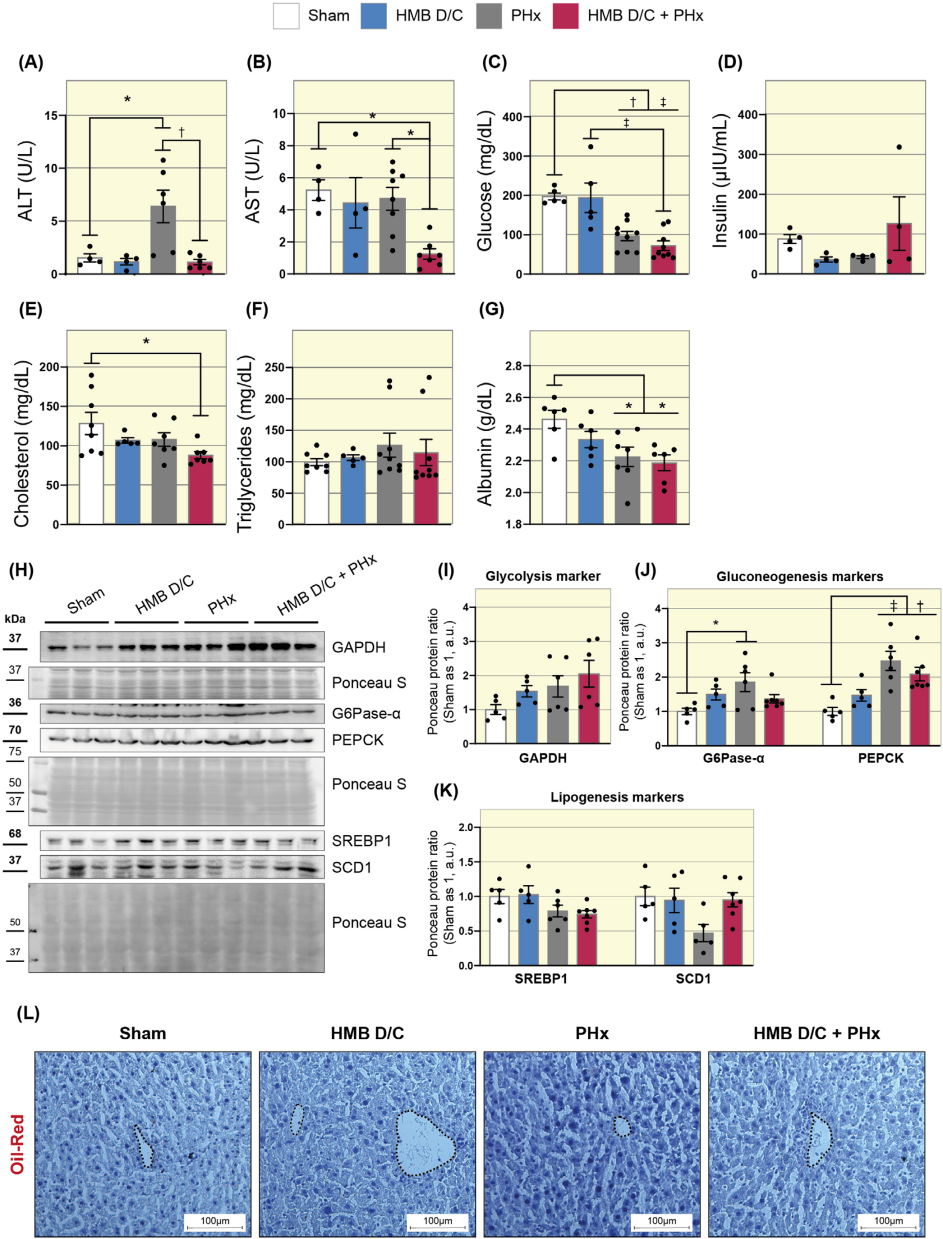
Prior supplementation with HMB reduces serum levels of hepatic damage markers after PHx. (A) Graph showing serum ALT levels (*n* = 4–6). (B) Graph showing serum AST levels (*n* = 4–8). (C) Graph showing serum glucose levels (*n* = 5–9). (D) Graph showing serum insulin levels (*n* = 4). (E) Graph showing serum cholesterol levels (*n* = 5–7). (F) Graph showing serum triglyceride levels (*n* = 5–9). (G) Graph showing serum albumin levels (*n* = 6). (H) Representative Western blot images of GAPDH, G6Pase‐α, PEPCK, SREBP1, and SCD1. (I) Representative graphs of the densitometric analysis of the protein content of GAPDH (*n* = 5–6). (J) Representative graphs of the densitometric analysis of the protein content of G6Pase‐α and PEPCK (*n* = 5–7). (K) Representative graphs of the densitometric analysis of the protein content of SREBP1 and SCD1 (*n* = 5–7). (L) Representative images of liver cross‐sections stained with Oil‐Red. The dashed lines mark the central veins. Images acquired with a 20× objective. Scale bar: 100 μm. Data are presented as mean ± SEM. The solid line represents One‐way ANOVA followed by Bonferroni's post hoc test: *p < 0.05, ^†^ *p* < 0.01, ^‡^ *p* < 0.001. Sham is set as 1; Ponceau S was used as a loading control.

Since we observed a discrepancy in liver mass between the PHx and HMB D/C + PHx groups, we evaluated liver damage using serum biochemical markers. We began by quantifying the activity of the ALT and AST enzymes, which indicate hepatocellular damage when observed at high levels in the bloodstream. The HMB D/C + PHx group exhibited a significant decrease in ALT (PHx 6.40 vs. HMB D/C + PHx 1.11 a.u.) and AST (PHx 4.69 vs. HMB D/C + PHx 1.24 a.u.) activities compared to the PHx group (Figure 3A,B). We also analyzed markers related to glucose and lipid metabolism. Both PHx groups exhibited lower blood glucose levels than the sham group (Figure 3C). However, serum insulin levels did not differ significantly between the groups (Figure 3D). The HMB D/C + PHx group demonstrated reduced serum total cholesterol levels relative to the sham group, which was not observed in the PHx group (Sham 128.34 vs. HMB D/C + PHx 88.06 a.u., Figure 3E). In the analysis of triglyceride levels, we have not found differences between the studied groups (Figure 3F). Finally, both PHx groups exhibited lower albumin levels than the sham group (Figure 3G). These findings suggest that hepatocellular injury was reduced by prior HMB supplementation.

We also investigated the content of proteins involved in central metabolic pathways in the liver. To this end, we began by investigating the effects of supplementation on hepatic glycemic metabolism, focusing on proteins associated with glycolysis and gluconeogenesis. We did not observe any changes in glyceraldehyde‐3‐phosphate dehydrogenase (GAPDH) protein levels between the study groups (Figure 3H,I). The PHx group exhibited an increase in glucose‐6‐phosphatase (G6Pase‐α) protein content compared to the sham group (Sham 1.0 vs. PHx 1.86 a.u.), which was not observed in the HMB D/C + PHx group (Figure 3H,J). Both groups undergoing ⅔ resection increased phosphoenolpyruvate carboxykinase (PEPCK) protein content compared to the sham group (Figure 3H,J). We also evaluated the content of proteins associated with lipogenesis, specifically sterol regulatory element‐binding protein 1 (SREBP‐1) and stearoyl‐CoA desaturase 1 (SCD1). However, we did not find significant effects on the levels of these proteins (Figure 3H,K), and consequently, we did not observe an accumulation of lipids in the tissue (Figure 3L). Together, our findings suggest minor alterations in gluconeogenic protein expression in the HMB‐supplemented group.

### 3.4 HMB Supplementation Reduces Ki‐67 and Increases Cyclin D1 Levels During Liver Regeneration

**FIGURE 4:**
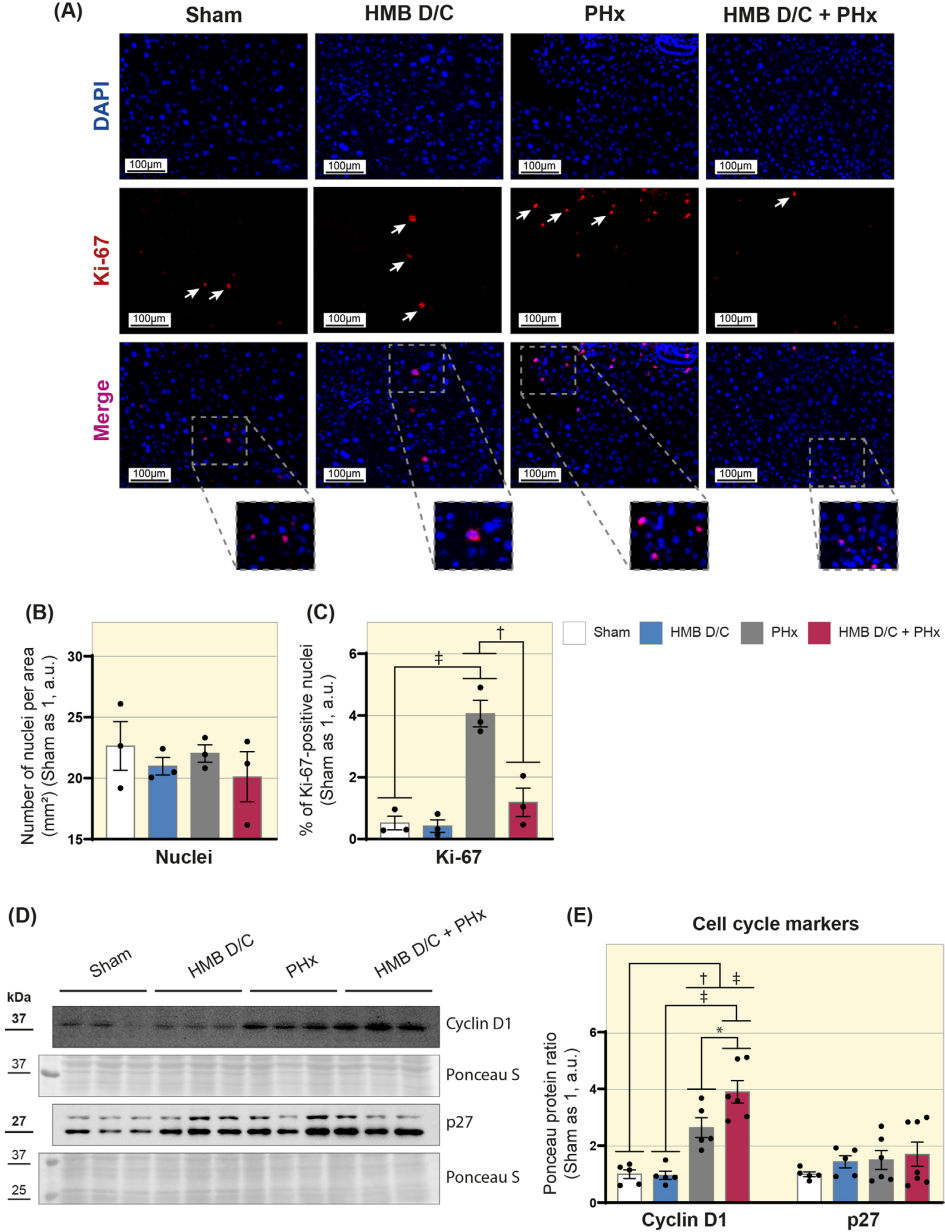
HMB supplementation changes Ki‐67 and Cyclin D1 levels during liver regeneration. (A) Representative images of Ki‐67 immunofluorescence in mouse liver cross‐sections. Images were acquired with a 40× objective. Scale bar: 100 μm. Arrows point to Ki‐67‐positive nuclei. (B) Graph showing the number of nuclei per area (mm^2^, *n* = 3). (C) Graph showing the percentage of Ki‐67‐positive nuclei (*n* = 3). (D) Representative Western blot images of Cyclin D1 and p27. (E) Representative graphs of the densitometric analysis of Cyclin D1 and p27 (*n* = 5–7). Data are presented as mean ± SEM. The solid dash represents one‐way ANOVA followed by Bonferroni's post hoc test: *p < 0.05, ^†^ *p* < 0.01, ^‡^ *p* < 0.001. Sham is set as 1; Ponceau S was used as a loading control.

The proportion between liver and body mass is strictly controlled during liver regeneration, but defects in the cell cycle progression can lead to an exaggerated accumulation of liver mass. In this context, we continued our analysis by investigating cell proliferation through immunofluorescence. We found no significant differences between the study groups in the number of nuclei per area (mm^2^, Figure 4A,B). However, our results showed an increase in the percentage of Ki‐67‐positive nuclei in the PHx group compared to the sham group (Sham 0.52 vs. PHx 4.06 a.u.). In contrast, the HMB D/C + PHx group reduced the percentage of Ki‐67‐positive nuclei compared to the PHx group and kept its levels similar to the sham group (PHx 4.06 vs. HMB D/C + PHx 1.19 a.u., Figure 4A,C).

Additionally, we analyzed the content of Cyclin D1, a crucial protein that facilitates the transition of cells from the G1 phase to the S phase of the cell cycle. The PHx and the HMB D/C + PHx groups exhibited increased Cyclin D1 protein content compared to the sham group. However, the HMB D/C + PHx group showed a significant increase compared to the PHx group (PHx 2.64 vs. HMB D/C + PHx 3.90 a.u., Figure 4D,E). Complementary, we analyzed the content of p27, a cyclin‐dependent kinase (CDK); however, we did not observe significant differences in the content of this protein between the study groups (Figure 4D,E). These results indicate that preoperative supplementation with HMB can regulate cell proliferation in the hepatic regenerative process induced by PHx.

### 3.5 Preoperative Supplementation With HMB Reduces the Parkin Signaling Independently of ATF4 During Liver Regeneration

**FIGURE 5:**
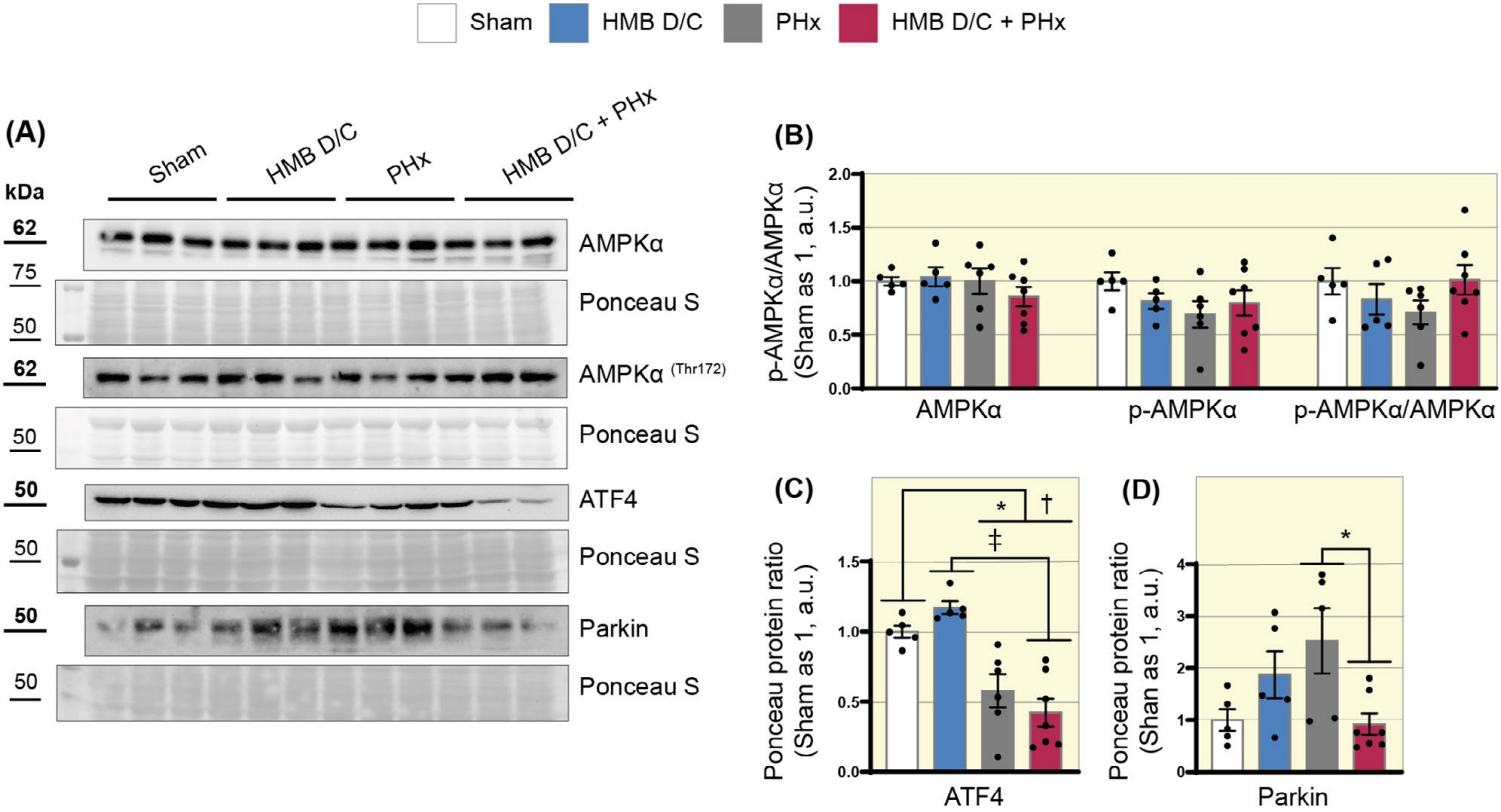
Preoperative supplementation with HMB reduces the ATF4‐Parkin signaling during liver regeneration. (A) Representative Western blot images of AMPKα, p‐AMPKα^(Thr172)^, Parkin, and ATF4. (B) Representative graphs of the densitometric analysis of AMPKα, p‐AMPKα, and the p‐AMPKα/AMPKα ratio (*n* = 5–7). (C) Representative graphs of the densitometric analysis of ATF4 (*n* = 5–7). (D) Representative graphs of the densitometric analysis of Parkin (*n* = 5–7). Data are presented as mean ± SEM. The solid dash represents One‐way ANOVA followed by Bonferroni's post hoc test: *p < 0.05, ^†^ *p* < 0.01, ^‡^ *p* < 0.001. Sham is set as 1; Ponceau S was used as a loading control.

Based on the evidence that HMB supplementation reduced hepatocellular injury, we analyzed proteins associated with cellular stress control. AMPKα (AMP‐activated protein kinase) functions as a metabolic sensor, responding to AMP/ATP ratio changes to restore energy balance during metabolic stress. Our results indicated no significant changes in AMPKα content, p‐AMPKα^(Thr172)^, or the p‐AMPKα/AMPKα ratio following PHx‐induced liver regeneration, regardless of prior supplementation (Figure 5A,B).

We also examined activating transcription factor 4 (ATF4), which modulates mitochondrial activity to mitigate excessive free radical production and cellular stress. Both groups undergoing ⅔ resection showed decreased ATF4 levels compared to the sham group without any effect of HMB supplementation on its content (Figure 5A,C). Among ATF4‐regulated proteins is Parkin, an E3 ligase critical for mitochondrial quality control, acting on mitophagy and mitochondrial biogenesis. Our findings revealed a significant reduction in Parkin protein content only in the HMB D/C + PHx group (PHx 2.52 vs. HMB D/C + PHx 0.92 a.u., Figure 5A,D). Therefore, these results suggest that preoperative HMB supplementation reduces Parkin content independently of ATF4 signaling.

ATF4 is activated by the mechanistic target of rapamycin complex 1 (mTORC1), assisting in maintaining cellular anabolism. Following PHx, liver regeneration relies not only on hepatocyte proliferation but also on the hypertrophic growth of existing liver cells. In this context, we evaluated the activation of markers related to protein synthesis pathways by examining the phosphorylation of Akt^(Ser473)^, 4E‐BP1^(Thr70)^, and Erk^(Thr202 and Tyr204)^. However, we did not observe statistically significant differences in the study groups (Figure S2). The absence of changes suggests that HMB alters the cell cycle without affecting protein synthesis markers during PHx‐induced stress.

### 3.6 Previous Supplementation With HMB Reduces MFN2 and DRP1 but Increases VDAC2 and Tom20 Levels During Liver Repair

**FIGURE 6:**
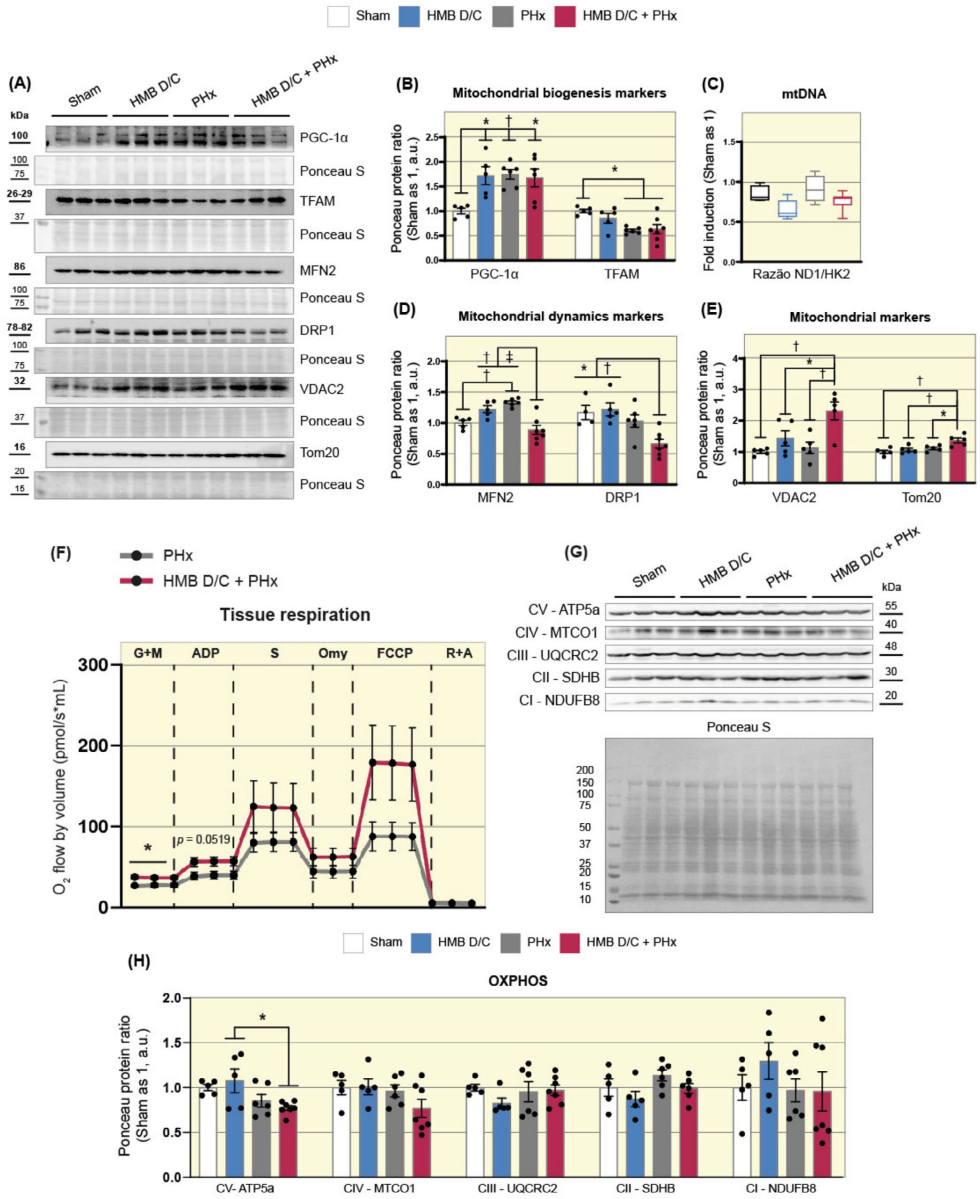
Previous supplementation with HMB reduces MFN2 and DRP1 but increases VDAC2 and Tom20 levels during liver repair. (A) Representative Western blot images of PGC‐1α, TFAM, MNF2, DRP1, VDAC2, and Tom20. (B) Representative graphs of the densitometric analysis of PGC‐1α and TFAM (*n* = 5–7). (C) Quantifying mtDNA by ND1/HK2 ratio through qPCR analysis in liver samples (*n* = 4–7). (D) Representative graphs of the densitometric analysis of MFN2 and DRP1 (*n* = 5–7). (E) Representative graphs of the densitometric analysis of VDAC2 and Tom20 (*n* = 5–7). Data are presented as mean ± SEM. The solid dash represents One‐way ANOVA followed by Bonferroni's post hoc test: *p < 0.05, †*p* < 0.01, ^‡^ *p* < 0.001. Sham is set as 1; Ponceau S was used as a loading control. (F) Graph showing the oxygen consumption rate (OCR) measured using high‐resolution respirometry (*n* = 5–6). The substrates used for the assay were: Malate + Glutamate; ADP; Succinate; Oligomycin; FCCP; and Rotenone + Antimycin. Data are presented as mean ± SEM. The asterisk (*) represents the Student's *t*‐test: *p < 0.05. (G) Representative Western blot images of ATP5a, MTCO1, UQCRC2, SDHB, and NDUFB8. (H) Representative graphs of the densitometric analysis of OXPHOS complexes. Data are presented as mean ± SEM. The solid dash represents One‐way ANOVA followed by Bonferroni's post hoc test: *p < 0.05.

Parkin is critical in mitochondrial homeostasis, mainly through mitophagy activation and mitochondrial biogenesis stimulation. Consequently, our study progressed to analyze proteins within these pathways. We observed that PGC‐1α, the primary transcription factor for mitochondrial biogenesis, exhibited increased content following HMB supplementation (Sham 1.0 vs. HMB 1.72 a.u.), surgery (Sham 1.0 vs. PHx 1.75 a.u.), and their combination (Sham 1.0 vs. HMB D/C + PHx 1.68 a.u., Figure 6A,B). PGC‐1α activation leads to mitochondrial transcription factor A (TFAM) synthesis, a mitochondrial transcription and replication regulator. However, both PHx groups showed reduced TFAM content compared to the sham group (Figure 6A,B). The ND1/HK2 ratio analysis of mtDNA content did not reveal significant differences between the studied groups (Figure 6C).

Given the Parkin role in mitochondrial quality control, including regulation of mitofusin 2 (MFN2; mitochondrial fusion) and dynamin‐related protein (DRP1; mitochondrial fission) expression, we examined these proteins. Our analysis indicated increased MFN2 protein content in the PHx group compared to the sham group (Sham 1.0 vs. PHx 1.33 a.u., Figure 6A,D). In contrast, the HMB D/C + PHx group displayed reduced MFN2 levels compared to the HMB D/C group (HMB D/C 1.22 vs. HMB D/C + PHx 0.89 a.u.) and PHx group (PHx 1.33 vs. HMB D/C + PHx 0.89 a.u., Figure 6A,D). Similarly, the HMB D/C + PHx group exhibited decreased DRP1 protein content compared to the sham group (Sham 1.0 vs. HMB D/C + PHx 0.67 a.u.) and HMB D/C group (HMB D/C 1.22 vs. HMB D/C + PHx 0.67 a.u.), a decrease not observed in the PHx group, where levels were comparable to the sham group (Figure 6A,D).

To assess the mitochondrial population, we examined voltage‐dependent anion channel 2 (VDAC2) and mitochondrial preprotein translocases of the outer membrane (Tom20). VDAC2 serves as a crucial channel for ADP/ATP transport, Ca^2+^ uptake, and regulation of apoptotic mechanisms, while Tom20 facilitates the import of mitochondrial proteins across the outer membrane. Both proteins showed significantly increased content in the HMB D/C + PHx group compared to other study groups (Figure 6A,E). These results suggest that prior HMB supplementation can modulate the protein content of mitochondrial transporters, as well as mitochondrial dynamics proteins.

To investigate whether the modulations induced by HMB in the content of proteins related to mitochondrial quality could result in improved tissue respiration after PHx, we assessed the oxygen consumption rate using high‐resolution respirometry (OROBOROS). The results indicated that the HMB D/C + PHx group exhibited a significant increase in basal tissue respiration (PHx 27.24 vs. HMB D/C + PHx 36.89 a.u., Figure 6F) compared to the PHx group. Additionally, when analyzing the protein levels of the five OXPHOS system complexes, we observed that the HMB D/C + PHx group modulated only Complex V (F1 ATP Synthase Alpha Subunit; ATP5a), leading to a reduction in its content compared to the HMB D/C group (HMB D/C 1.08 vs. HMB D/C + PHx 0.77 a.u., Figure 6G,H). These data suggest that HMB pre‐conditioning slightly enhances hepatic basal respiration following PHx, a phenomenon potentially mediated by an increase in mitochondrial efficiency rather than an increase in the content of the OXPHOS machinery.

Finally, we proceeded with our investigation by examining the levels of proteins associated with autophagy, the process responsible for maintaining cellular health through the degradation of cellular components such as proteins, protein aggregates, and damaged organelles. The protein sequestosome 1 (p62) acts as a critical receptor that targets proteins for degradation via the proteasome or autophagosome. The microtubule‐associated protein light chain 3 (LC3) is crucial for autophagosome formation and extension. In our study, the HMB D/C + PHx group exhibited reduced p62 content compared to the PHx group (PHx 1.53 vs. HMB D/C + PHx 0.91 a.u., Figure S3). However, no significant differences were observed between the groups when evaluating the LC3‐I and LC3‐II fractions, nor the LC3‐II/I ratio (Figure S3). The decrease in p62 levels in the HMB D/C + PHx group suggests a potential increase in autophagic flux in this group.

### 3.7 Prior HMB Supplementation Increases mtDNA Content and Enhances Mitochondrial Respiration Following Successive Liver Injury

**FIGURE 7:**
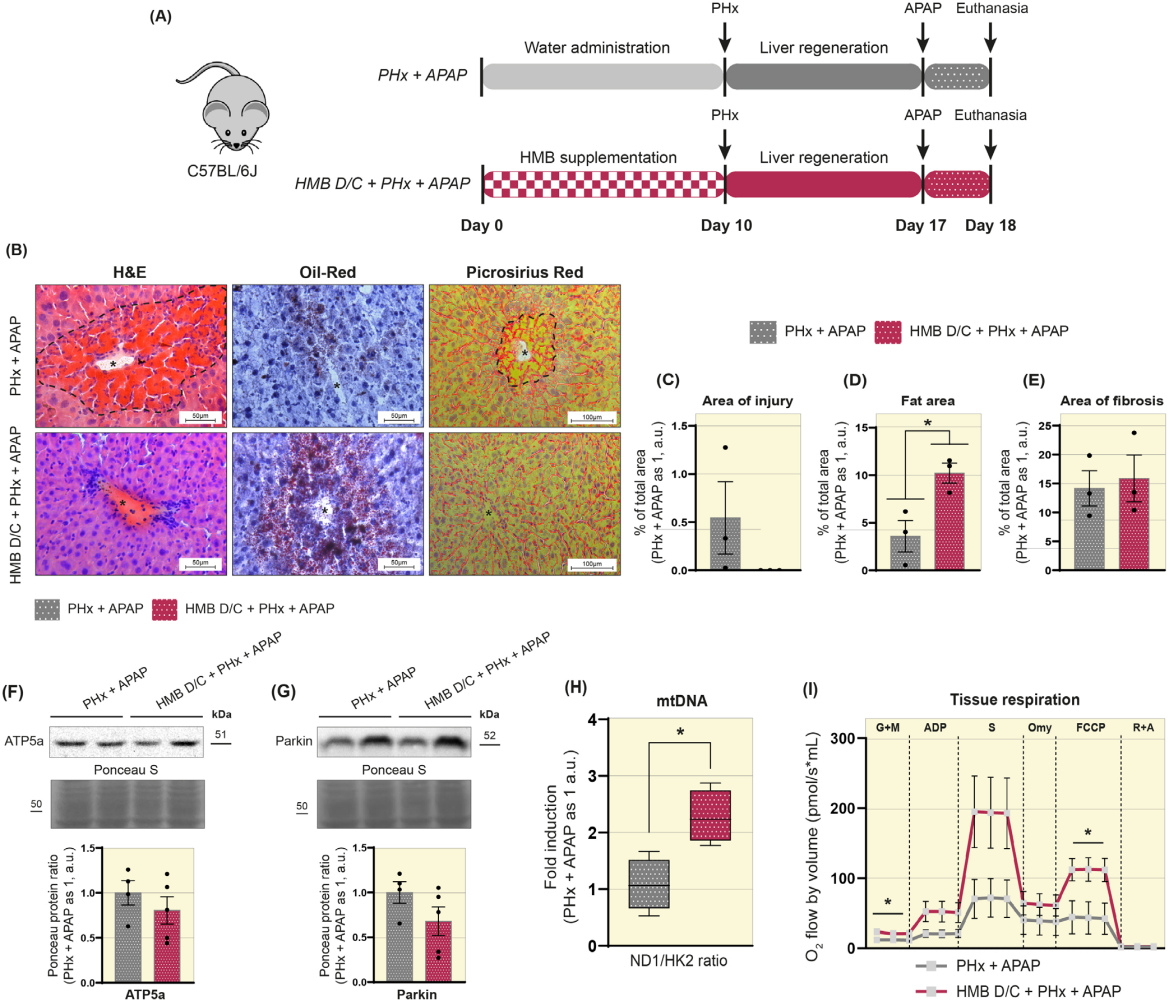
Prior HMB supplementation increases mtDNA content and enhances mitochondrial respiration following successive liver injury. (A) A diagram illustrating the administration of APAP overdose after 7 days of liver regeneration in mice subjected to partial hepatectomy. (B) Representative images of liver cross‐sections stained with H&E, Oil‐Red, and Picrosirius Red. The dashed line represents the lesion area. The asterisk marks the central vein. Images acquired with 20× and 40× objectives. Scale bar: 50 μm and 100 μm. (C) Graph showing total area of injury (*n* = 3). (D) Graph showing total fat area (*n* = 3). (E) Graph showing total area of fibrosis (*n* = 3). (F) Representative Western blot images and quantification of ATP5a (*n* = 4–5). (G) Representative Western blot images and quantification of Parkin (*n* = 4–5). (H) Quantifying mtDNA by ND1/HK2 ratio through qPCR analysis in liver samples (*n* = 4–5). (I) Graph showing the oxygen consumption rate (OCR) measured using high‐resolution respirometry (*n* = 4–5). The substrates used for the assay were: Malate + Glutamate; ADP; Succinate; Oligomycin; FCCP; and Rotenone + Antimycin. Data are presented as mean ± SEM. The solid dash represents the Student's *t*‐test: *p < 0.05.

Given that an increase in basal respiration alone does not necessarily reflect an improvement in overall mitochondrial function, and also to understand whether HMB supplementation could be beneficial to liver capacity post‐PHx, we imposed an additional challenge on the liver tissue. To this end, on the 7th day of regeneration, we administered an overdose of APAP via gavage and, after 24 h, euthanized the mice by anesthetic overdose (Figure 7A). In the PHx + APAP group, histological analysis revealed the presence of centrilobular lesions, as evidenced by H&E staining, which was not observed in the HMB D/C + PHx + APAP group (Figure 7B). However, the measurement of the injury area did not show differences between the groups studied (Figure 7C). Oil Red O staining indicated an accumulation of centrilobular lipid droplets, especially in the HMB D/C + PHx + APAP group, which showed a significant increase in the fat area compared to the PHx + APAP group (PHx + APAP 3.59 vs. HMB D/C + PHx + APAP 10.24 a.u., Figure 7B,D). Picrosirius Red staining identified connective tissue distributed throughout the liver tissue in both groups (Figure 7B), and the measurement of the fibrosis area did not reveal significant differences (Figure 7E).

We investigated the protein content of previously evaluated markers and found no significant differences between the study groups regarding the levels of ATP5a and Parkin (Figure 7F,G). However, despite maintaining the same ATP5a levels, the HMB D/C + PHx + APAP group showed increased mtDNA copy number compared with the PHx + APAP group (PHx + APAP 1.08 vs. HMB D/C + PHx + APAP 2.28 a.u., Figure 7H) as well as enhanced tissue respiration under both basal conditions (PHx + APAP 11.87 vs. 21.78 a.u.) and maximal respiratory capacity (PHx + APAP 43.48 vs. 112.39 a.u., Figure 7I). These results suggest that HMB supplementation can promote adaptations in the mitochondrial capacity of liver tissue, enabling it to respond positively even after exposure to successive stresses.

## 4 Discussion

The efficiency of the hepatic regenerative process is essential for preserving liver function and, by extension, the body's homeostasis. In this context, mitochondrial functionality plays a critical role in this process by providing the energy necessary for tissue restructuring and supporting the survival and proliferation of liver cells. We hypothesized that HMB supplementation before PHx could modulate mitochondrial quality control pathways and, consequently, influence tissue repair. Our results suggest that preoperative HMB supplementation can modulate some mitochondrial markers, particularly those involved in mitochondrial dynamics, by reducing the protein levels of Parkin, Mfn2, and DRP1 while increasing the content of mitochondrial transporters such as VDAC2 and Tom20. As a result, the HMB‐supplemented group demonstrated increased mtDNA content and enhanced tissue respiration even after a second injury from an APAP overdose.

It has been demonstrated that oral HMB supplementation, especially in skeletal muscle tissue, can stimulate mitochondrial biogenesis and protein synthesis pathways, including the AKT/mTOR pathway [28, 29]. Therefore, as a first step, we evaluated the effects of HMB supplementation per se on the protein content of key markers of protein synthesis and mitochondrial biogenesis. In our study, the dose used is equivalent to the one used in humans, expected to be sufficient to elicit effects. However, the absence of changes in these markers may be attributable to the small sample size and the duration of supplementation. It is possible that pronounced effects would have been observed with a longer period of HMB administration in the mice. However, oral HMB supplementation usually exerts its effects under stressful conditions [30], increasing muscle mass and strength under pathological conditions like sarcopenia, cancer cachexia, and aging [31, 32, 33, 34]. With this in mind, we next investigated its effects during hepatic stress induced by PHx.

The ability of hepatocytes to replicate in response to liver tissue loss is a fundamental aspect of liver regeneration. Normally, cell proliferation decreases 5–7 days post‐PHx as liver mass is restored. Our findings indicate that the HMB‐supplemented group maintained liver mass at levels comparable to the sham group, whereas the PHx group exhibited an increase beyond this parameter. Disruptions in liver cell cycle progression can lead to an imbalance in the liver mass‐to‐body mass ratio, with prolonged proliferation resulting in excessive liver mass accumulation [35].

Following the idea that HMB could regulate the cell cycle, our study shows that preoperative HMB supplementation increases Cyclin D1 protein levels, which may be associated with decreased Parkin levels in this group. Parkin has been implicated in inhibiting cell proliferation in lung and colorectal cancers by activating p53, upregulating p21, and reducing Cyclin D1 levels [36]. However, in the context of liver regeneration, reduced Cyclin D1 levels may be insufficient to support adequate liver cell proliferation, potentially arresting hepatocytes in the G1 phase of the cell cycle [37, 38]. Thus, combining reduced Parkin and increased Cyclin D1 under HMB supplementation suggests that hepatocytes could be engaged in cell cycle progression. To support this idea, the HMB supplementation reduced the percentage of Ki‐67‐positive cells to levels observed in the sham group. In contrast, the PHx group exhibited elevated cell proliferation during the final regeneration phase. Ki‐67 is a marker of active cell division commonly used to assess tumor cell proliferation [39, 40]. The termination phase of regeneration is crucial for preventing the regenerating liver from undergoing tumorigenesis and proliferating excessively, which could lead to it exceeding its ideal mass [35, 41]. The effects of liver resection on the growth of intra‐ and extrahepatic tumors remain controversial. Picardo et al. [42] demonstrated that cytokines and growth factors such as TGF‐α and TGF‐β stimulate both hepatocyte proliferation and liver regeneration, yet these same mediators can also promote tumor growth. Although DNA synthesis typically ceases approximately 48 h after resection, tumor cell proliferation often persists beyond this point, which aligns with our observations. Additionally, factors such as the extent of hepatectomy (particularly major resections), post‐PHx immunosuppression, and disruption of Kupffer cell function contribute to tumor development, underscoring its multifactorial nature.

In our study, macroscopic anatomical examination revealed the growth of a hepatic mass near the surgical incision site, particularly in the PHx group. This mass was noticeably smaller in the group previously supplemented with HMB, which may reflect the effects of the supplement on cell‐cycle progression described earlier. Moreover, the characteristics of this lesion were consistent with a hepatic adenoma, especially in the PHx group. Hepatic adenomas are benign tumors typically presenting as yellowish, smooth, rounded, well‐defined, and pseudoencapsulated masses. They are usually solitary and may reach sizes large enough to be detected on physical examination in humans, with their risk of malignant transformation increasing with the tumor size [43]. Three main subtypes of hepatic adenomas have been described, each defined by distinct morphological and molecular features [44]. Inflammatory adenomas exhibit substantial inflammatory infiltrate, consistent with the histopathological findings in the HMB D/C + PHx group. In contrast, adenomas harboring HNF‐1α mutations are usually highly steatotic due to absent L‐FABP expression. Supporting this phenotype, the PHx group displayed marked lipid accumulation at the site of mass growth, unlike the HMB‐supplemented mice. It is important to note that we did not perform immunohistochemical analyses of specific adenoma markers; therefore, our interpretation remains speculative. However, the combined macroscopic and histopathological findings suggest that preoperative HMB supplementation may help regulate cell proliferation, thereby preventing excessive hepatic mass accumulation after resection.

As demonstrated, the progression of the hepatocyte cell cycle can influence the final liver size. However, a volumetric increase in this organ does not always equate to functional improvement. In our results, we observed a significant increase in the total laminin‐stained area in the PHx group, which was not observed in the previously supplemented group. Laminin is a major component of the extracellular matrix (ECM) in liver tissue and is closely associated with the proliferation and differentiation of hepatic progenitor cells (HPCs) [45]. Under physiological conditions, laminin is scarcely detectable in the liver in the absence of active fibrogenesis and is typically restricted to the portal areas [46], as also observed in the sham group of our study. However, laminin content increases during hepatic fibrosis, thereby promoting the differentiation of HPCs into cholangiocytes and impairing the efficient generation of mature hepatocytes [47]. The accumulation of laminin in the space of Disse, in addition to the physiological presence of type IV collagen, is associated with the pathogenesis of hepatic sinusoidal capillarization during fibrogenesis. This pathological process is characterized by the formation of a continuous basement membrane, accompanied by thickening of sinusoidal endothelial cells, reduced fenestration, development of a basement lamina within the space of Disse, and perisinusoidal collagen deposition. Together, these alterations establish a filtration barrier between the sinusoids and hepatocytes, ultimately contributing to hepatic dysfunction [46]. Laminin content in the supplemented group did not differ significantly from the sham group, despite an observed numerical increase. This group also exhibited reduced serum levels of the liver enzymes ALT and AST. During liver regeneration, these enzymes typically increase in the serum due to parenchymal alterations, and their levels decrease as the liver regenerates [48, 49]. This group responded positively to a secondary injury induced by an overdose of APAP, suggesting that HMB supplementation may contribute to reducing the hepatocellular injury.

Furthermore, the loss of liver mass induces transient steatosis, characterized by the accumulation of triglycerides, fatty acids, and cholesterol esters. The mobilization of lipids into hepatocytes serves as an energy substrate for cell proliferation and can be used to form cell membranes [50]. This reduces the need to redirect energy from other cellular activities toward hepatic regenerative processes, thereby preventing the liver's metabolic function impairment [51]. This steatosis typically resolves within 2 days post‐resection [52], which may explain the absence of changes in lipogenesis markers or lipid droplets in our study. However, 24 h after a second injury, we observed a greater accumulation of lipid droplets in the HMB‐supplemented group. In myocytes, HMB can also promote lipid accumulation and mitochondrial biogenesis [53]. These findings suggest that the liver tissue of this group maintains its ability to regulate lipid metabolism after a second injury.

Given that preoperative supplementation with HMB reduced hepatocellular damage, we evaluated the protein content of ATF4, a transcription factor that activates the integrated stress response. We found that PHx reduces ATF4 levels, regardless of whether HMB supplementation is present. Endoplasmic reticulum (ER) stress can induce ATF4 expression through translational upregulation via the PERK‐eIF2α pathway, which is associated with inflammatory responses [54]. However, we did not detect any differences in the levels of inflammatory markers, including SOD2, NF‐κB, IL‐6, or IL‐10, which may be attributable to the specific regeneration time point analyzed. On the other hand, ATF4 expression and activation are also regulated by mTORC1 [55]. Therefore, we investigated the impact of oral HMB supplementation on the protein synthesis pathway in the context of liver regeneration induced by ⅔ resection. Our analysis did not reveal a significant effect on protein synthesis markers in this context. Since tissue repair initially involves hepatocyte hypertrophy, our focus on the final phase of regeneration may have overlooked critical early‐stage changes related to protein synthesis, which can be considered a limitation of our study. Finally, the ATF4, in the nucleus, can transcriptionally regulate genes involved in autophagy and mitophagy [56], like Parkin. Parkin is an E3 ligase that plays a critical role in maintaining mitochondrial homeostasis by regulating the degradation of dysfunctional mitochondria through mitophagy. Our results showed that PHx reduced levels of ATF4, but only the HMB‐supplemented group exhibited decreased levels of Parkin. These findings indicate that the reduction in Parkin levels results from HMB supplementation during the recovery from PHx independently of ATF4.

Parkin also promotes mitochondrial biogenesis by ubiquitinating and facilitating the proteasomal degradation of PARIS, a suppressor of PGC‐1α [57]. Here, we observed an upregulation of PGC‐1α while we found a downregulation of TFAM, without significant changes in mtDNA content. Mitochondrial biogenesis is a key process in tissue repair and the recovery of cellular function following stress, primarily driven by the upregulation of the transcriptional coactivator PGC‐1α. PGC‐1α is regulated by AMPKα, an energy sensor [58]; however, we did not detect significant differences in the levels of AMPKα, phosphorylated AMPKα, or the p‐AMPKα/AMPKα ratio in our study. PGC‐1α regulates several downstream targets, including the nuclear respiratory factors NRF1 and NRF2, which play essential roles in mitochondrial biogenesis. These transcription factors control the expression of genes involved in mtDNA transcription and replication. Since we did not assess NRF1 and NRF2 expression, we cannot determine whether these factors were available to promote TFAM expression, encoded by NRF1, or to change mtDNA copy number. In addition, estrogen‐related receptor α (ERRα) is a coactivator effector of PGC‐1α and is associated with the induction of genes involved in mitochondrial biogenesis and oxidative phosphorylation [59]. Shires et al. [60] demonstrated that Parkin can translocate to the nucleus during cellular stress induced by hypoxia, where it interacts with and ubiquitinates ERRα. Since PHx induces hypoxia and cellular stress, Parkin may be similarly translocated to the nucleus under these conditions and act on ERRα. This could explain the increase in PGC‐1α content without corresponding changes in TFAM expression or mtDNA copy number. Unfortunately, we did not assess the subcellular localization of Parkin, and this remains a limitation in our study that can be investigated in the future.

Looking for other mitochondrial dynamics players, we observed a decrease in the content of DRP1 and MFN2 in the HMB‐supplemented group. Mitochondrial fission, DRP1 as an important marker, allows dysfunctional mitochondria to be eliminated by mitophagy, helping to avoid excessive generation of reactive oxygen species (ROS) [61]. In addition, in pathophysiological conditions when cells are under stress, ROS increases the activation of DRP1, leading to mitochondrial fragmentation and phagocytosis [62]. However, excessive mitochondrial fission can result in mitochondrial dysfunction. Hepatic ischemia–reperfusion injury (IRI) is common after hepatic surgeries, particularly PHx and liver transplantation, as it involves a prolonged ischemic insult followed by the restoration of blood perfusion. This process can lead to poor liver transplant outcomes due to excessive mitochondrial fission. Huang et al. [63] demonstrated that inhibition of DRP1 SUMOylation by the augmenter of liver regeneration (ALR) reduced mitochondrial fragmentation and preserved liver function following IRI. The elevated mitochondrial fragmentation in cholestatic liver disease was associated with increased oxidative stress, while the inhibition of mitochondrial fission reduced apoptosis, fibrosis, and liver injury [64]. APAP toxicity is also associated with mitochondrial fragmentation and inhibition of mitochondrial fusion proteins [58]. With this in mind, our findings showed a decrease in DRP1 levels in the HMB‐supplemented group, a change not observed in the PHx group. The HMB‐supplemented group also demonstrated increased basal tissue respiration both before and after successive liver injuries, as well as the ability to mobilize lipids and elevate mtDNA copy number following APAP overdose, suggesting preservation of its mitochondrial function.

In contrast, under stress conditions, such as nutrient deprivation, metabolic sensors can trigger mitochondrial elongation and fusion (MFN2 as a key marker), allowing mitochondria to remain connected for longer periods to increase ATP production [65]. Our results showed a decrease in MFN2 levels compared to the PHx group, which increased its content compared to the sham group. However, mitochondrial elongation can facilitate the reintegration of damaged mitochondria into the mitochondrial network, which can reduce the efficiency of eliminating defective mtDNA and compromise mitochondrial functionality [66]. Post‐PHx, mtDNA replication and transcription occur in the early phase of regeneration, where an adequate number of mtDNA copies is crucial for maintaining mitochondrial function and ATP production to sustain the regenerative process and cell proliferation [67]. Our limited period analysis, focused on the final phase of the regenerative process, could explain our lack of any modulation in the quantification of mtDNA content between the groups. Interestingly, following a second injury induced by APAP overdose, the HMB‐supplemented group showed an increase in mtDNA copy number, an important response associated with liver regeneration, which was not observed in the PHx group.

Mitochondrial quality control is also the target of studies in HMB supplementation models. C2C12 myotubes treated with HMB have increased expression of mitochondrial regulatory genes, such as PGC‐1α and NRF‐1 [68]. In 3T3‐L adipocytes, HMB increased basal respiration, maximal respiration, and ATP production [69]. Our data pointed to HMB supplementation to an increase in VDAC2, a crucial voltage‐dependent channel in the mitochondrial membrane that controls the transport of ADP and ATP, Ca^2+^ uptake, and regulates apoptotic mechanisms. Several aspects of mitochondrial function are modulated by Ca^2+^ transport; thus, the different VDAC isoforms can interact with cytosolic proteins or with cytoskeletal elements to regulate OMM permeability to Ca^2+^ and promote energy efficiency. On high‐resolution respirometry, the previously supplemented group exhibited an increase in basal respiration. Zhao et al. [70] further discovered that Ca^2+^ influx into mitochondria during mitosis, especially during metaphase‐anaphase transition, can play a key role in cell cycle progression. Thus, our data indicated that pre‐operative HMB supplementation could regulate mitochondrial quality proteins to preserve mitochondrial stability in the liver during the regeneration process, aiding in proper progression in the cell cycle and preserving the tissue against hepatocellular damage. On this point, our second injury model highlighted the protective effect of HMB supplementation over mitochondrial capacity before the PHx.

**FIGURE 8:**
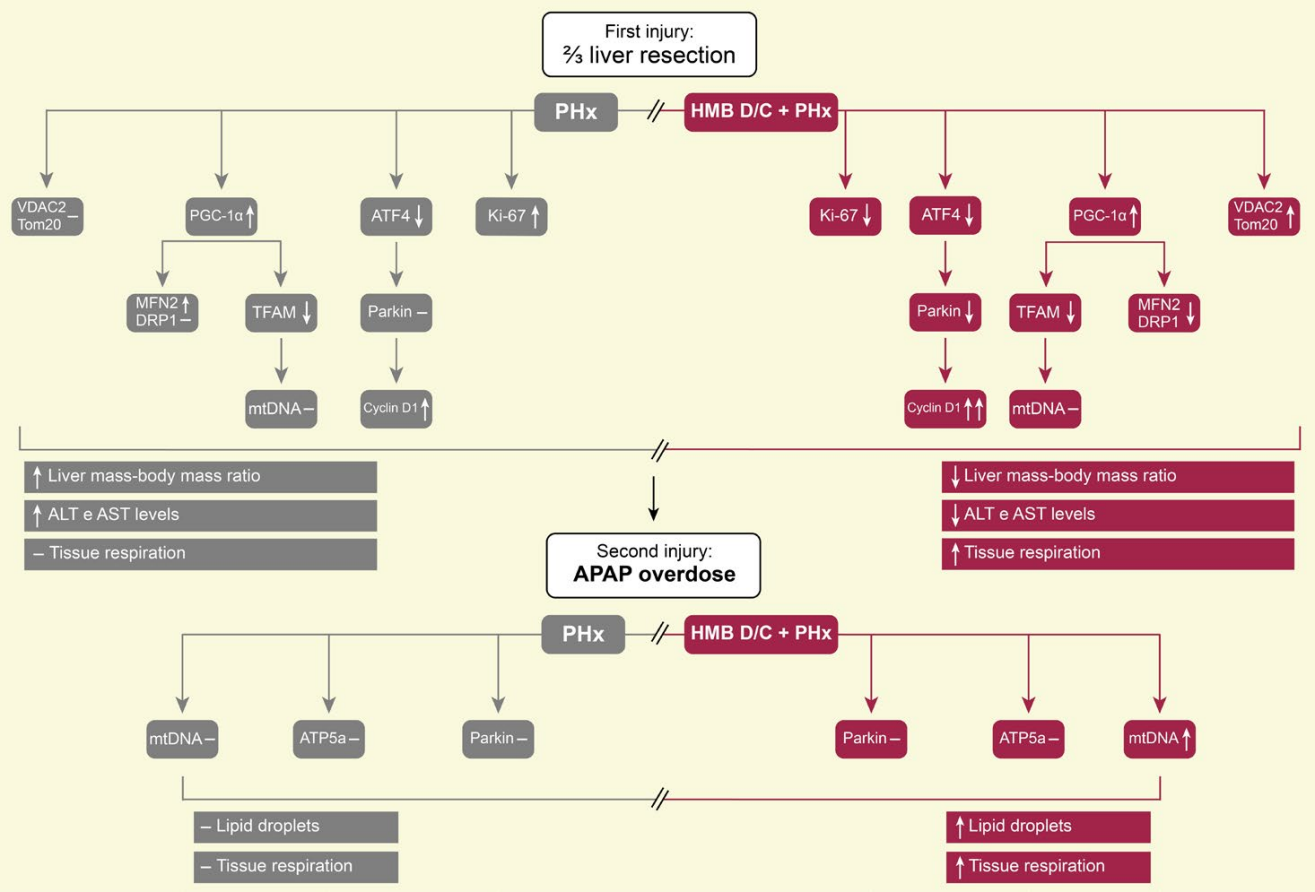
Summary of the effects of preoperative supplementation with HMB on liver regeneration. PHx increased the content of cell cycle markers, specifically Cyclin D1 and Ki‐67, and reduced ATF4 levels without affecting its transcriptional target, Parkin. Additionally, PHx increased PGC‐1α, the primary transcription factor associated with mitochondrial biogenesis, although its downstream target, Tfam, was decreased. No significant differences were observed in mtDNA content or the mitochondrial markers VDAC2 and Tom20. Regarding mitochondrial dynamics, PHx increased Mfn2 levels. These findings were accompanied by an increase in the liver mass/body mass ratio and elevated ALT enzyme activity, indicating hepatocellular injury. However, preoperative HMB supplementation increased Cyclin D1 levels while reducing Ki‐67. The HMB decreased both ATF4 and Parkin levels. Similar to the PHx group, HMB increased PGC‐1α content and reduced Tfam levels but did not alter mtDNA content. HMB also increased the levels of mitochondrial markers VDAC2 and Tom20 and reduced both mitochondrial dynamics markers, Mfn2 and DRP1. Additionally, HMB increases tissue respiration. As a result, HMB supplementation reduced the liver mass/body mass ratio and lowered ALT and AST enzyme activity. Consequently, when subjected to a second injury induced by APAP overdose, HMB supplementation increased the mtDNA content, lipid droplet area, and mitochondrial capacity of the tissue, changes that were not observed in the group that did not receive prior supplementation before PHx. The “–” represents the absence of modulation; the arrow pointing up represents an increase; the arrow pointing down represents a decrease.

The administration of high doses of APAP induces the formation of the metabolite N‐acetyl‐p‐benzoquinoneimine (NAPQI), which depletes cellular stores of hepatic glutathione and forms complexes with cellular proteins, especially in the mitochondria, increasing the production of reactive oxygen species and causing mitochondrial dysfunction [71, 72]. Our experimental groups showed similar levels of ATP5a; however, the HMB‐supplemented group exhibited a higher mtDNA content and increased respiratory capacity, indicating a greater efficiency in energy production. Mitochondria have been shown to play an essential role in recovery after APAP‐induced injury. For example, the accumulation of dysfunctional mitochondria due to inhibition of mitophagy exacerbates APAP‐induced hepatotoxicity [73], while the synthesis of new mitochondria is important for tissue recovery [74]. In parallel, we need to consider HMB as a substrate for HMG‐CoA biogenesis, and consequently, HMG‐CoA reductase to produce mevalonic acid, a precursor of coenzyme Q, also known as ubiquinone [75]. The increase in ubiquinone is frequently related to increasing the antioxidant capacity and improving the mitochondrial capacity of different cell types [76]. Thus, we can suggest that HMB supplementation could increase mitochondrial health by improving the antioxidant capacity of liver tissue previously, for the PHx. This idea is reinforced by the reduction of Parkin levels, an increase of VDAC2, the ability to produce more mtDNA, and even the less necessity of ATP5 levels observed here after the ⅔ resection. Our major findings are summarized in Figure 8.

We investigated the effects of HMB pre‐operative supplementation over the PHx, focused on the final recovery, and the impact on mitochondrial quality control pathways. We have discussed several rational explanations for our findings, mainly considering the potential effects of HMB as a regulator of mitochondrial proteins and biogenesis. However, it is important to highlight that we did not analyze short‐term effects, for example, the possible regulation of stellate hepatic cells in the first hours of PHx, or the inflammatory response. To our knowledge, our study is the first to evaluate the ability of pre‐operative HMB supplementation to change mitochondrial health even after a second challenge after PHx in a mouse model. Further studies are needed to clarify the ability of HMB supplementation to improve mitochondrial health in other hepatic conditions or clinical demands.

In conclusion, our study demonstrates that preoperative HMB supplementation can reduce hepatocellular injury after PHx by maintaining mitochondrial quality and improving the liver antioxidant capacity during the regenerative process. Our findings suggest that HMB supplementation regulates mitochondrial dynamics by decreasing Parkin, Mfn2, and DRP1 levels while increasing VDAC2 and Tom20. The positive effects of HMB supplementation on liver and mitochondrial function were further corroborated by our findings after a second injury induced by APAP overdose. The HMB‐supplemented group exhibited an increase in mtDNA content and mitochondrial capacity following successive injuries, which is crucial for tissue recovery.

## Author Contributions

**A. L. Vieira‐da‐Silva:** conceptualization, investigation, formal analysis, visualization, writing – original draft, writing – review and editing, data curation, methodology. **M. V. Esteca:** data curation, formal analysis, visualization, methodology, investigation, writing – review and editing. **F. A. Silva:** investigation, methodology – surgical technique, writing – review and editing. **I. A. Divino:** data curation, formal analysis, visualization, methodology, writing – review and editing. **F. S. Carneiro:** methodology, investigation, data curation. **E. R. Ropelle:** formal analysis, data curation, supervision. **A. S. Torsoni:** formal analysis, data curation, writing – review and editing, supervision. **I. L. Baptista:** original draft, conceptualization, data curation, formal analysis, visualization, methodology, investigation, supervision, project administration, writing – review and editing, funding acquisition, resources.

## Funding

This research was supported by the São Paulo Research Foundation (FAPESP grant #2019/12236‐5 and #2023/01903‐6). A.L. Vieira‐da‐Silva received the FAPESP Fellowship (#2021/05588‐2). This work was also financed in part by the National Council for Scientific and Technological Development (CNPq—grants #403549/2021‐3 and #420265/2018‐0) and also financed in part by the Coordenação de Aperfeiçoamento de Pessoal de Nível Superior—Brasil (CAPES)—Finance Code 001 (no. 88887.827297/2023‐00).

## Conflicts of Interest

The authors declare no conflicts of interest.

## Supporting information


**Figure S1:** Representative images of liver cross‐sections stained with hematoxylin and eosin (H&E) and Picrosirius Red. Representative Western blots and densitometry for SOD2, NF‐κB, IL‐6, and IL‐10.
**Figure S2:** Representative Western blots and densitometry for Akt, Akt^(Ser473)^, 4E‐BP1, 4E‐BP1^(Thr70)^, ERK1/2, and ERK1/2^(Thr202/Tyr204)^.
**Figure S3:** Representative Western blots and densitometry analyses for p62 and LC3‐I/II.
**Table S1:** Primary antibodies used in Western blot and immunofluorescence techniques.
**Table S2:** Secondary antibodies used in Western blot and immunofluorescence techniques.

## Data Availability

The data that support the findings of this study are available on request from the corresponding author. The data are not publicly available due to privacy or ethical restrictions.
