# Supplementary material for: Preoperative Beta‐Hydroxy‐Beta‐Methyl‐Butyrate Supplementation Reduces Mitochondrial Dynamics Proteins and Preserves Hepatic Mitochondrial Function After Partial Hepatectomy in Mice
Source: Acta Physiol (Oxf). 2026 Mar 29;242(5):e70204. doi: 10.1111/apha.70204 (PMC13033621; doi:10.1111/apha.70204)
Supplement: Supplementary file 1 — Figure S1: Representative images of liver cross‐sections stained with hematoxylin and eosin (H&E) and Picrosirius Red. Representative Western blots and densitometry for SOD2, NF‐κB, IL‐6, and IL‐10. Figure S2: Representative Western blots and densitometry for Akt, Akt(Ser473), 4E‐BP1, 4E‐BP1(Thr70), ERK1/2, and ERK1/2(Thr202/Tyr204). Figure S3: Representative Western blots and densitometry analyses for p62 and LC3‐I/II. Table S1: Primary antibodies used in Western blot and immunofluorescence techniques. Table S2: Secondary antibodies used in Western blot and immunofluorescence techniques. [file APHA-242-e70204-s001.docx]

***Picrosirius red staining:***

Slides containing histological sections underwent a graded rehydration series, immersing them sequentially in ethanol solutions of decreasing concentrations (100%, 90%, 70%, 50%, and 30%) followed by distilled water for 1 min each step. Subsequently, the sections were stained with hematoxylin for 5 min and then rinsed in running tap water for 2 min. Afterward, the slides were incubated in a Picrosirius Red staining solution (0.1 g of Direct Red 80 in a saturated aqueous solution of picric acid ー 1.3 g in 100 mL of distilled water) for 40 min at 37°C in an incubator. This was followed by 2 acid-washing steps, each involving immersion in an acetic acid solution (1 mL of acetic acid in 200 mL of distilled water) for 5 min. The slides were then subjected to a dehydration sequence, which involved sequential immersion in distilled water and increasing concentrations of ethanol (30%, 50%, 70%, 90%, and 100%), followed by xylene, each step lasting 1 min. After air-drying, the slides were mounted using Entellan™ (Sigma Aldrich®) as the mounting medium. The images were acquired with a light microscope (Leica®) using 20× and 40× objectives. The area of associated connective tissue was counted using ImageJ software (Research Services Branch, National Institute of Mental Health). In this analysis, an average of 5 photographs per animal from different regions and sections of the liver were used.

**Table 1. Primary antibodies**

| **Antibody** | **Code** | **Brand** | **Concentration** |
| --- | --- | --- | --- |
| p-ERK1/2 | #4370 | Cell Signaling | 1:1000 |
| ERK1/2 | #9102 | Cell Signaling | 1:1000 |
| p-4E-BP1 (Thr 70) | #9455s | Cell Signaling | 1:1000 |
| 4E-BP1 | #9452s | Cell Signaling | 1:1000 |
| p-AKT (ser 473) | sc-33437 | Santa Cruz | 1:500 |
| AKT | sc-8312 | Santa Cruz | 1:500 |
| PGC-1α | Pa5-38022 | Invitrogen | 1:1000 |
| Parkin | #2132s | Cell Signaling | 1:1000 |
| Laminin | L9393 | Sigma-Aldrich | 1:100 |
| SOD2 | #13141 | Cell Signaling | 1:1000 |
| NF-kB p65 | #8242 | Cell Signaling | 1:1000 |
| IL-6 | P620 | Invitrogen | 1:1000 |
| IL-10 | ARC9102 | Invitrogen | 1:1000 |
| GAPDH | sc-25778 | Santa Cruz | 1:1000 |
| G6Pase-α | sc-25840 | Santa Cruz | 1:500 |
| PEPCK | sc-32879 | Santa Cruz | 1:500 |
| SREBP1 | GTX79299 | GeneTex | 1:1000 |
| SCD1 | #2794 | Cell Signaling | 1:1000 |
| Ki-67 | ab15580 | Abcam | 1:50 |
| Cyclin D1 | #2978s | Cell Signaling | 1:1000 |
| p27 | sc-528 | Santa Cruz | 1:1000 |
| p-AMPKα (Thr 172) | #50081s | Cell Signaling | 1:1000 |
| AMPKα | #2532s | Cell Signaling | 1:1000 |
| ATF4 | #11815s | Cell Signaling | 1:1000 |
| TFAM | Pa5-29571 | Invitrogen | 1:1000 |
| MFN2 | ab124773 | Abcam | 1:1000 |
| DRP1 | #8570s | Cell Signaling | 1:1000 |
| VDAC2 | ab37985 | Abcam | 1:1000 |
| Tom20 | #42406s | Cell Signaling | 1:1000 |
| OXPHOS | ab110413 | Abcam | 1:1000 |
| SQSTM1/p62 | #23214s | Cell Signaling | 1:1000 |
| LC3A/B | #4108s | Cell Signaling | 1:1000 |

**Table 2. Secondary antibodies**

| **Antibody** | **Code** | **Brand** | **Concentration** |
| --- | --- | --- | --- |
| Peroxidase AffiniPure  Donkey Anti-Goat IgG (H+L) | 705-035-003 | Jackson | 1:5000 |
| Peroxidase AffiniPure Goat Anti-Rabbit IgG (H+L) | 111-035-003 | Jackson | 1:5000 |
| Peroxidase AffiniPure Goat Anti-Mouse IgG (H+L) | 115-035-003 | Jackson | 1:5000 |
| Alexa Fluor 594 AffiniPure Donkey Anti-Rabbit IgG (H+L) | 711-585-152 | Jackson | 1:250 |

**Supplementary figure legends:**

**
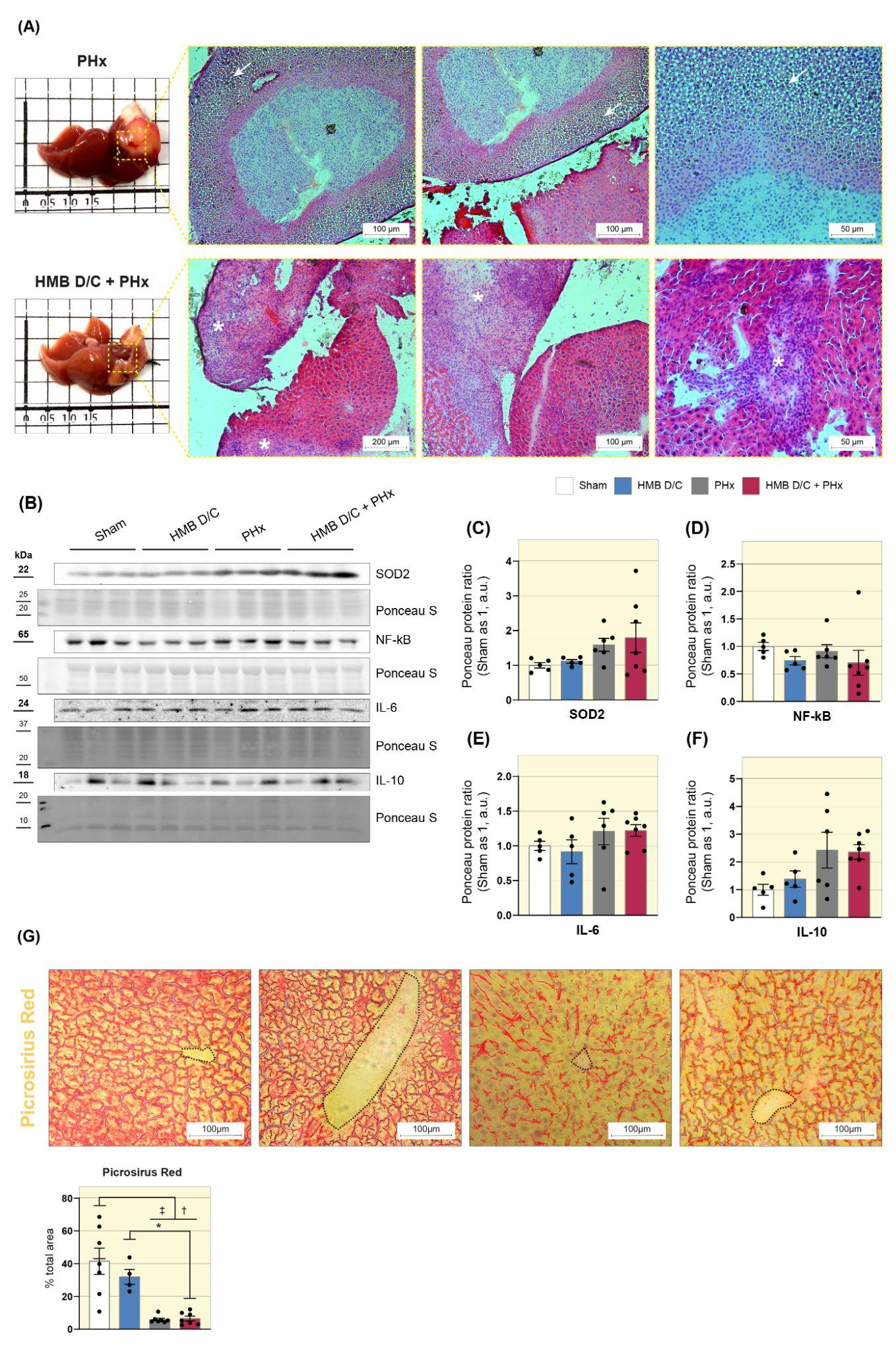
**

**Figure S1. (A)** Representative images of liver cross-sections stained with H&E. The arrows mark the fat accumulation, and the asterisks indicate the inflammatory infiltrate. Images acquired with a 10×, 20×, and 40× objective. Scale bar: 200, 100, and 50 μm. **(B)** Representative Western Blot images of SOD2, NF-kB, IL-6, and IL-10. **(C)** Graphical representations of SOD2 content (n = 5-7). **(D)** Graphical representations of NF-kB content (n = 5-7). **(E)** Graphical representations of IL-6 content (n = 5-7). **(F)** Graphical representations of IL-10 content (n = 5-7). Data are presented as mean ± SEM. Sham is set as 1; Ponceau S was used as a loading control. **(G)** Representative images of liver cross-sections stained with Picrosirius Red. The dashed lines mark the central veins. Images acquired with a 20× objective. Scale bar: 100 μm. Graph showing the total area of connective tissue content (n = 4-7). Data are presented as mean ± SEM. The solid line represents One-way ANOVA followed by Bonferroni's post hoc test: ✝ *p* < 0.01, ‡ *p* < 0.001. Sham is set as 1.

**
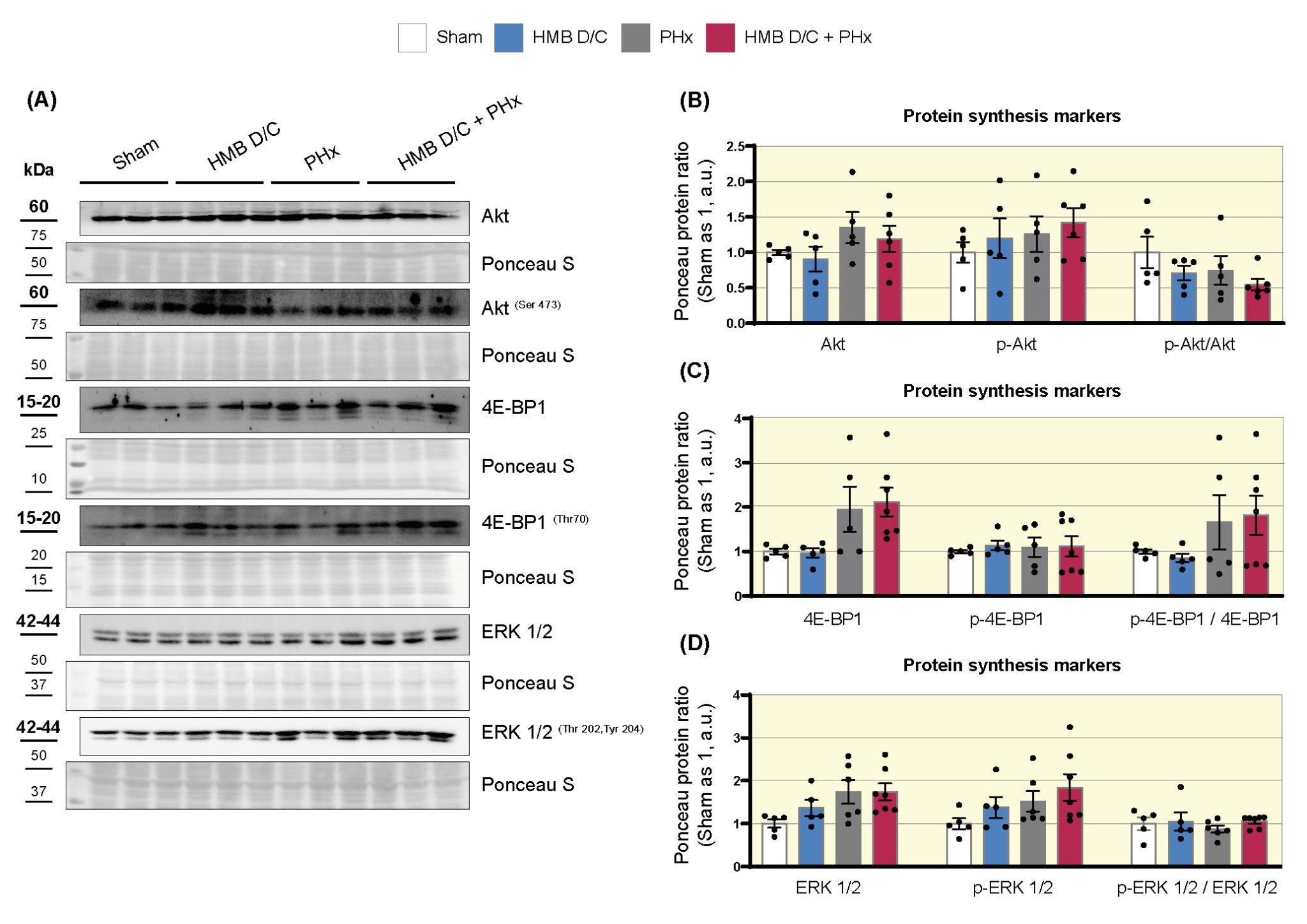
**

**Figure S2. (A)** Representative Western Blot images of Akt, p-Akt^(Ser473)^, 4E-BP1, p-4E-BP1^(Thr70)^, ERK 1/2, and p-ERK 1/2^(Thr202, Tyr204)^. **(B)** Graphical representation of Akt, p-Akt, and the p-Akt/Akt ratio (n = 5-6). **(C)** Graphical representations of the protein content of 4E-BP1, p-4E-BP1, and the p-4E-BP1/4E-BP1 ratio (n = 5-7). **(D)** Graphical representations of the protein content of ERK 1/2, p-ERK 1/2, and the ERK 1/2/p-ERK 1/2 ratio (n = 5-7). Data are presented as mean ± SEM. The solid dash represents One-way ANOVA followed by Bonferroni's post hoc test: * *p* < 0.05, ✝ *p* < 0.01, ‡ *p* < 0.001. Sham is set as 1; Ponceau S was used as a loading control.

**
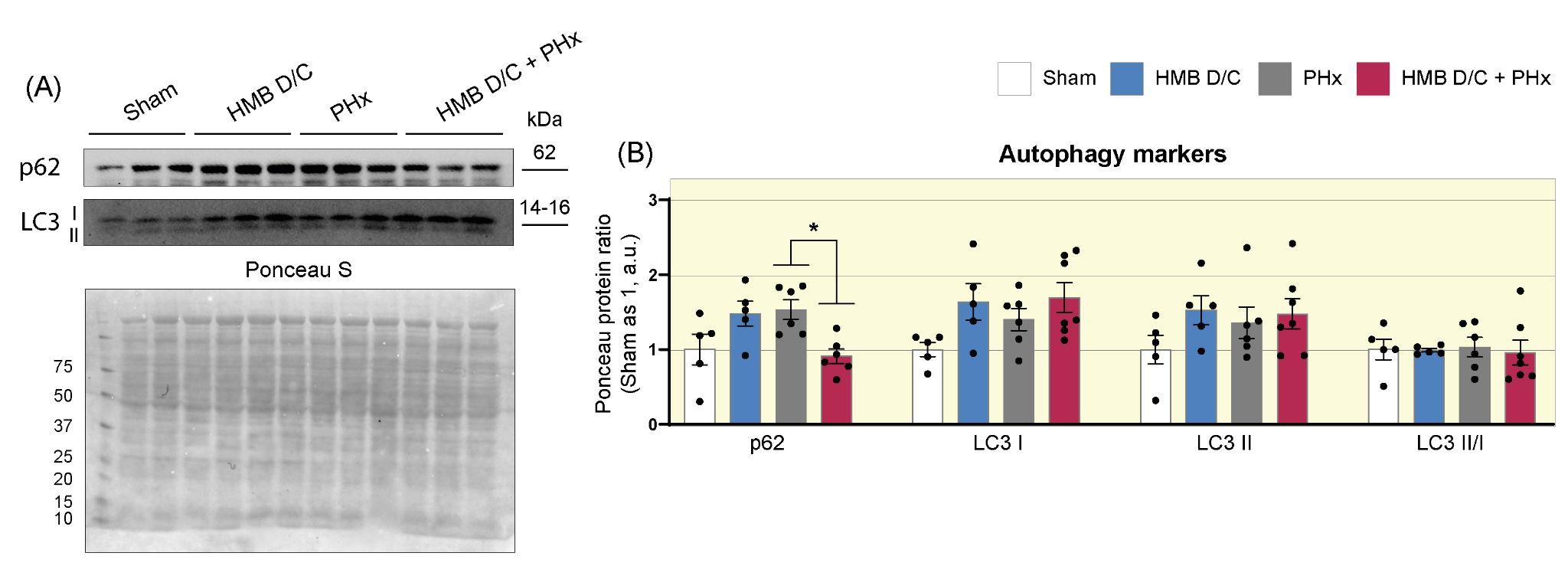
**

**Figure S3.** **(A)** Representative Western Blot images of p62 and LC3. **(B)** Graphical representations of the protein content of p62, LC3 I, LC3 II, and the LC3 II/I ratio (n = 5-7). Data are presented as mean ± SEM. The solid dash represents One-way ANOVA followed by Bonferroni's post hoc test: * *p* < 0.05. Sham is set as 1; Ponceau S was used as a loading control.
